# Adenosine‐to‐inosine editing of miR‐200b‐3p is associated with the progression of high‐grade serous ovarian cancer

**DOI:** 10.1002/1878-0261.70106

**Published:** 2025-08-08

**Authors:** Magdalena Niemira, Anna Skwarska, Karolina Chwialkowska, Agnieszka Ostrowska, Gabriela Sokolowska, Anna Zeller, Anna Erol, Andrzej Eljaszewicz, Bartosz Hanczaruk, Anna Michalska‐Falkowska, Agnieszka Tarasik, Joanna Reszec‐Gielazyn, Pawel Knapp, Marcin Moniuszko, Adam Kretowski

**Affiliations:** ^1^ Clinical Research Centre Medical University of Bialystok Poland; ^2^ Comprehensive Cancer Center Albert Einstein College of Medicine Bronx New York USA; ^3^ Centre of Regenerative Medicine Medical University of Bialystok Poland; ^4^ Biobank Medical University of Bialystok Poland; ^5^ Department of Medical Pathology Medical University of Bialystok Poland; ^6^ University Oncology Centre University Clinical Hospital in Bialystok Poland; ^7^ Department of Allergology and Internal Medicine Medical University of Bialystok Poland; ^8^ Department of Endocrinology, Diabetology and Internal Medicine Medical University of Bialystok Poland

**Keywords:** ADAR, A‐to‐I RNA editing, high‐grade serous ovarian cancer, microRNAs

## Abstract

Deamination of adenosine to inosine (A‐to‐I) in double‐stranded microRNAs (miRNAs) has been demonstrated to affect their function as suppressors or oncogenes in various cancers. Nevertheless, the functional impact of miRNA editing in high‐grade serous ovarian cancer (HGSOC) remains largely unexplored. Here, we identified A‐to‐I editing in miRNAs in 60 HGSOC tissues and 48 ovarian tissues received in nononcological procedures using small RNA sequencing (RNA‐Seq). To investigate the functional impact of A‐to‐I modifications, we tested the effect of edited RNA mimics and small interfering RNA (siRNA)‐mediated downregulation of the RNA‐editing enzyme double‐stranded RNA‐specific editase Adar (ADAR1) on cell proliferation, migration and three‐dimensional (3D) growth of HGSOC cells *in vitro*. Tumour suppressor miR‐200b‐3p was the most overedited miRNA in HGSOC tumours, and the increased editing level was associated with statistically significant worse overall survival (OS). Mechanistically, in contrast to wild‐type miRNA, edited miR‐200b‐3p promoted cell proliferation, migration and formation of 3D spheroids. Loss of function of *ADAR1* profoundly repressed proliferation, migration and 3D growth of HGSOC cells. RNA‐Seq and Gene Set Enrichment Analysis (GSEA) analysis revealed that, whereas wild‐type miR‐200b‐3p induced the apoptosis pathway, edited miR‐200b‐3p substantially inhibited cell‐cycle‐related pathways. Bioinformatic prediction revealed that edited miR‐200b‐3p gained the function to repress the expression of new targets, including tumour suppressor MAX interactor 1, dimerisation protein (*MXI1*), which was associated with a statistically significantly worse OS time in HGSOC patients. Our study reports the potential contribution of edited miR‐200b‐3p in HGSOC progression, and highlights its potential as a new therapeutic target.

## Graphical abstract

A‐to‐I editing of miRNAs, particularly miR‐200b‐3p, contributes to HGSOC progression by enhancing cancer cell proliferation, migration and 3D growth. The edited form is linked to poorer patient survival and the identification of novel molecular targets. These findings highlight its potential as a biomarker and therapeutic target in ovarian cancer.

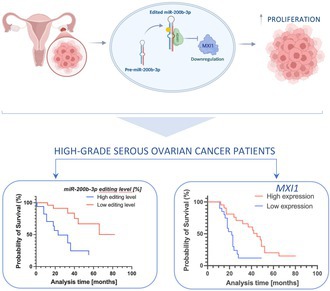

**Abbreviations**
3′UTR: 3′untranslated region
3D: three‐dimensional
8‐aza: 8‐azaadenosine
ADAR1: double‐stranded RNA‐specific editase Adar
A‐to‐I: adenosine‐to‐inosine
BMI: body mass index
CPM: counts per million
DEGs: differentially expressed genes
ED: edited
EMT: epithelial‐mesenchymal transition
FDR: false discovery rate
GSEA: gene set enrichment analysis
H&E: haematoxylin and eosin
HGSOC: high‐grade serous ovarian cancer
IHC: immunohistochemistry
MCC: maximum clique centrality
miRNA: microRNA
MTT: 3‐(4,5‐diethylthiazol‐2‐yl)‐2,5‐diphenyltetrazolium bromide
MXI1: MAX interactor 1, dimerisation protein
NC: negative control
ncRNAs: non‐coding RNAs
NES: normalised enrichment score
OS: overall survival
PBS: phosphate‐buffered saline
PPI: protein–protein interactions
RIN: RNA integrity number
RISC: RNA‐induced silencing complex
RPM: reads per million
SD: standard deviation
siRNA: small interfering RNA
STR: short tandem repeat
TMM: trimmed mean of M values
TNM: tumour‐node‐metastasis
WB: western blot
WT: wild‐type
ZEB1: zinc finger E‐box‐binding homeobox 1

## 1 Introduction

RNA editing is a post‐transcriptional process that modifies the primary RNA transcripts, creating new information in the RNA that is not encoded directly in the DNA [1]. Adenosine‐to‐inosine (A‐to‐I) RNA editing is the most abundant form of RNA editing in humans, accounting for ~90% of all RNA editing events [2]. This reaction is mediated by a double‐stranded RNA‐specific editase Adar (ADAR) family of enzymes through hydrolytic deamination of the adenine base [3]. Upon this modification, inosines are recognised as guanosines and base‐pair with cytosines [4]. MicroRNAs (miRNAs), as small noncoding RNAs (ncRNAs), can also undergo A‐to‐I editing, altering their structure and biogenesis by modifying Drosha or Dicer cleavage sites or RNA‐induced silencing complex (RISC) loading [5, 6].

Altered A‐to‐I RNA editing in miRNA has been extensively studied in cancer. Analyses of miRNA editing events in 20 cancer types identified 19 editing ‘hotspots’ correlated with key molecular drivers such as *TP53*, *BRAF*, *NRAS*, *HRAS*, *CDH1*, *PIK3CA* and *MAP3K1* [7]. A broader study of > 13 000 tumour samples across 38 cohorts from the TCGA confirmed these findings [8].

A‐to‐I editing in miRNAs significantly impacts their function by shifting target specificity, disrupting precise base pairing and redirecting miRNA to new mRNA targets [9]. For example, edited miR‐379‐5p inhibits proliferation and promotes apoptosis in diverse tumour cell lines by targeting adhesion G protein‐coupled receptor E5 (*CD97*), while edited miR‐3144‐3p facilitates liver cancer progression through induction of Musashi RNA‐binding protein 2 (*MSI2*) and suppresses solute carrier family 38 member 4 (*SLC38A4*) [10, 11]. Similarly, edited miR‐200b‐3p promotes thyroid and breast cancer progression by reducing binding to *ZEB1* (zinc finger E‐box‐binding homeobox 1), a target for wild‐type miR‐200b‐3p [12, 13]. In melanoma, decreased *ADAR1* expression leads to diminished A‐to‐I editing of miR‐455‐5p and miR‐378‐3p, resulting in reduced levels of the tumour suppressor *CPEB1* (cytoplasmic polyadenylation element binding protein) and increased expression of *PARVA* (alpha‐parvin), thereby promoting cell invasion [14, 15]. In breast cancer, edited miR‐200b‐3p suppresses LIFR (leukaemia inhibitory factor receptor) expression, facilitating enhanced cell migration and invasion [7]. In glioblastoma, reduced ADAR2 activity decreases the editing of miR‐376‐5p and miR‐589‐3p, which contributes to tumour aggressiveness via upregulation of *AMFR* (autocrine motility factor receptor), the target of the edited miR‐376a‐5p, and *ADAM12* (ADAM metallopeptidase domain 12), the target of the edited miR‐589‐3p [16, 17].

Here, we investigated the functional consequences of A‐to‐I miRNA editing in high‐grade serous ovarian cancer (HGSOC) patients and preclinical models. Small RNA sequencing in 106 patients revealed that miRNA was edited most frequently in the tested HGSOC specimens compared with healthy controls. Significantly, a higher level of miR‐200b‐3p editing correlated with a decreased survival rate. Mechanistically, the miR‐200b‐3p editing increased the aggressiveness of ovarian cancer cells, proliferation, migration and formation of 3D spheroids in *in vitro* models. Furthermore, the editing of miR‐200b‐3p caused a shift in its targetome, leading to decreased mRNA translation of several tumour suppressors, including *MXI1*, a key antagonist of the c‐MYC oncogene, whose underexpression is correlated with a poor survival rate in HGSOC patients. Importantly, our study highlights the potential prognostic and therapeutic benefits of edited miR‐200b‐3p in HGSOC.

## 2 Materials and methods

### 2.1 Study cohort

The study was approved by the Ethics Committee of the Medical University of Bialystok (R‐I‐002/357/2014, R‐I‐002/600/2019 and APK.002.171.2021) and conducted according to the Declaration of Helsinki. Before sample collection, written informed consent for specimen collection was obtained from all participants. Samples were obtained between 2016 and 2022 from patients undergoing surgical treatment for HGSOC in the Clinical Hospital in Bialystok, collected by the Biobank at the Medical University of Bialystok, with high standards of strict biobanking procedures described further by Niklinski *et al*. [18]. miRNA and total RNA sequencing analyses were performed using 108 specimens, including 60 from patients with HGSOC and 48 from 48 control ovarian tissues received in nononcological procedures. Clinicopathological characteristics, such as age, body mass index (BMI), tumour stage according to FIGO Ovarian Cancer Staging and tumour‐node‐metastasis (TNM) staging system, and survival data were available. The basic clinical parameters describing each of the cohorts are summarised in Table 1.

**Table 1:** Clinicopathological characteristics of HGSOC patients and healthy subjects enrolled in the study.

Characteristics	HGSOC patients (*n* = 60)	Ovarian tissues (*n* = 48)	*P*
Age [y.o]	61.2 ± 10.6 (38–86)	58.6 ± 11.3 (34–77)	0.19^t^
Overall Survival [mo.]	30.2 ± 19.9 (8.0–81.9)	–	
**FIGO stages**, *n* (%)			
FIGO I	7 (6.7%)	–	
FIGO II	2 (3.4%)	–	
FIGO III	36 (62.1%)	–	
FIGO IV	10 (17.2%)	–	
Unknown	5 (10.3%)	–	
**TNM stages**, *n* (%)			
1	7 (11.7%)	–	
2	2 (3.3%)	–	
3	46 (76.7%)	–	
Unknown	5 (8.3%)	–	
***N*—lymph nodes**, *n* (%)			
0	9 (15.0%)	–	
1	46 (76.7%)	–	
Unknown	5 (8.3%)	–	
***M*—metastasis**, *n* (%)			
0	50 (83.3%)	–	
1	10 (8.4%)	–	
Unknown	5 (8.3%)	–	

t denotes t‐test.

### 2.2 Sample size estimation

Based on our previous experiments and pilot data, we calculated the minimal number of primary HGSOC samples (tumour or normal) required to detect twofold differences in the relative miRNA or mRNA expression at true positive detection powers of 80% and 90% [19]. We have used the RNA‐Seq Power R Package, applying the statistics data covering obtained real depth and coefficients of variation per each group separately because tumour and normal tissue differ in terms of interindividual variations, as well as mRNA and miRNA median lengths differ based on literature and databases [20, 21]. Each calculation was performed separately for the tumour and normal tissue groups for mRNA and miRNA. To obtain 80% detection power, 12 samples per mRNA tissue group and 20 samples per miRNA tissue group were required, whereas 16 samples in mRNA groups and 27 samples per miRNA group were required to obtain 90% detection power. Our patient groups comprised 60 (tumour tissue) samples and 48 (ovarian tissues received in non‐oncological procedures).

### 2.3 miRNA profiling by next‐generation sequencing

Total RNA with miRNA fraction was extracted from tissue samples using the *mir*Vana™ Isolation Kit (ThermoFisher Scientific, Waltham, MA, USA) according to the manufacturer's instructions. RNA concentration, purity and integrity were assessed by Qubit (Invitrogen, Waltham, CA, USA) and Tape Station (Agilent Technologies, Santa Clara, CA, USA). Small RNA‐Seq libraries were constructed from 1 μg of total RNA with an RNA integrity number (RIN) > 8 using the Illumina TruSeq Small RNA Preparation Kit (Illumina, San Diego, CA, USA). Indexed libraries were pooled and sequenced on the NovaSeq6000, generating 50‐bp single‐end reads (1 × 50 bp). Sequencing reads were subjected to quality checks before and after trimming and filtering steps using *fastQC* (Babraham Institute, Cambridge, UK) and *multiQC* [22]. Raw reads were trimmed from adapter sequences with *cutadapt* and only reads that contained adapters were kept for further analysis. After adapter trimming, reads were filtered further with cutadapt based on quality and length so that only reads of 16–28 bp were kept. Reads were then collapsed considering unique read sequences to the same mature miRNA cluster, and the observed counts were estimated across samples. miRNAs were aligned using bowtie and annotated to the human miRBase v22 and *miRge 3.0* package [23]. Raw miRNA counts were used as input for DE analysis that was conducted in the *R* statistical environment. Raw count data were filtered for lowly expressed miRNAs based on the corresponding Counts per Million (CPM) values. Counts were normalised using a weighted trimmed mean of the log expression ratios (trimmed mean of *M* values—TMM) [24]. Data were transformed using *voom* [25] and linear modelling with empirical Bayes moderation was applied using the *limma* package [26]. to assess the differential expression of miRNAs. *P*‐values were corrected for multiple testing using the Benjamini–Hochberg False Discovery Rate (FDR) procedure.

### 2.4 Identification of A‐to‐I editing

The mapped output file was subjected to identifying reads containing A to G changes representing A‐to‐I editing in mature miRNA using the *miRge 3.0* package [23]. Reads were aligned with the last two nucleotides trimmed in the 3' end, and one mismatch was allowed. Unique best hits were demanded, and for the retained miRNA/isomiR reads, their sequence was screened for A to G changes apart from terminal 5 bp based on the binomial test considering the expected sequencing error rate. *P*‐values were corrected for multiple testing using the Benjamini–Hochberg False Discovery Rate (FDR) procedure. The A‐to‐I editing level was defined as the ratio of the mapped reads with changed nucleotides relative to the total mapped reads for each miRNA. The putative A‐to‐I editing events were subjected to the following exclusion criteria to improve specificity based on the presence of A/G variations in the miRNAs and respective families: miRNAs located in the repeat elements that are prone to false positives; expression level of the canonical miRNA was less than one Read per Million (RPM); miRNAs with A to G changes were multimapping. A‐to‐I editing events were further analysed based on the respective percentage and expression level at the RPM scale.

### 2.5 RNA‐seq sample preparation and sequencing

Total RNA (1 μg) with an RNA Integrity Number (RIN) ≥ 8 was used to prepare sequencing libraries using the TruSeq Stranded Total RNA Library Prep Gold kit (Illumina). Indexed libraries were pooled and sequenced on the Illumina NovaSeq 6000 platform, generating 2 × 100 bp paired‐end reads. Quality control of raw and processed reads was performed using FastQC (Babraham Institute) and multiQC [22]. Adapter trimming and filtering of low‐quality bases were carried out with BBduk (DOE Joint Genome Institute, Berkeley, CA, USA). Cleaned reads were aligned to the GRCh38 human reference genome using the STAR aligner [27], which accounts for splice junctions and was run with quantMode enabled to obtain gene‐level counts. Gene annotations were based on the Ensembl (European Bioinformatics Institute) release version (v)GRCh38.98. Raw gene counts were used as input for the identification of differentially expressed genes (DEGs). Raw count data were filtered to identify genes expressed at low levels, < 10 raw counts in the smallest library, considering corresponding equivalent counts per million (CPM) values. The counts were normalised using a weighted trimmed mean of the log expression ratios (trimmed mean of *M* values—TMM). The data were transformed for linear modelling using the voom tool. Linear modelling and empirical Bayes moderation were applied using the limma package to assess the differential expression of genes [25]. Multiple FDR testing was applied to correct *P*‐values. This analytical workflow has been previously described in similar studies [28, 29].

### 2.6 qPCR

miRNAs and two reference miRNAs were profiled using the miRCURY LNA SYBR® Green PCR Kit (Qiagen, Hilden, Germany). Reference miRNAs with stable expression, hsa‐103‐3p and hsa‐199b‐5p, were selected using the NormFinder algorithm [30]. The miRCURY LNA RT Kit (Qiagen) was used for the reverse transcription reaction for the miRNA assay. Assay IDs are assembled in Table S1. Transcription High Fidelity cDNA Synthesis Kit (Roche, Basel, Switzerland) and KiCqStart SYBR® Green qPCR ReadyMix™ (Sigma‐Aldrich, St. Louis, MO, USA) were used for mRNA analysis. The primer sequences are assembled in Table S1. The temperature profile of the qPCR was as follows: 2 min at 95 °C and 45 cycles: 10 s at 95 °C and 60 s at 56 °C (for miRNA) and 60 s at 60 °C (for mRNA). Amplification was performed using LightCycler 480 (Roche). Subsequently, PCR threshold cycles (*C*_t_) of the tested miRNA/mRNA and reference miRNA/mRNA were determined for the tested samples and the calibrator. The relative expression for each miRNA/mRNA was calculated with PCR efficiency correction [31]. Efficiency (*E*) was calculated from the slopes of the calibration curve according to the equation: *E* = 10 (−1/slope). Reactions with amplification efficiency below 1.6 were removed. The relative expression ratio of a target miRNA was computed based on its PCR efficiencies (*E*) and the *C*_t_ value difference (Δ) of unknown group samples (test) *versus* the control group (Δ *C*_t_ control‐test). The relative calculation was based on the MEAN *C*_t_ of the experimental group. This analytical workflow has been previously described in similar studies [32].

### 2.7 Western blot analysis

Cells were lysed in RIPA Lysis and Extraction buffer (ThermoFisher Scientific) with the protease inhibitor (Roche). Protein concentration was assessed with the BCA kit (ThermoFisher Scientific) according to the manufacturer's instructions. Proteins (50 μg) were separated by SDS/PAGE and transferred to PVDF membranes using the Trans‐Blot Turbo Blotting System (Bio‐Rad, Hercules, CA, USA). The following antibodies were used: anti‐MXI1 (#PA5‐68756; ThermoFisher Scientific), anti‐ADAR1 (#EPR7033; Abcam, Cambridge, UK) and anti‐B‐actin‐HRP conjugated (#ab8227; Abcam). The blots were developed with the SuperSignal West Femto Kit (ThermoFisher Scientific) and visualised on the ChemiDoc Imaging System (Bio‐Rad).

### 2.8 Cell culture and 3D spheroid cultures

The human high‐grade ovarian cancer OVCAR3 (ATCC® HTB‐161) (RRID:CVCL_0465) and CAOV3 (ATCC® HTB‐75) (RRID:CVCL_0201) cell lines were purchased from the American Type Culture Collection (ATCC, Manassas, VA, USA). OVCAR3 cells were maintained in RPMI 1640 medium, while CAOV3 cells were grown in DMEM. All media were supplemented with 10% FBS (ATCC), 100 U·mL^−1^ penicillin and 100 μg·mL^−1^ streptomycin (Merck, Darmstadt, Germany). Cells were kept at 37 °C under 5% CO_2_. Cell line identity was confirmed by the Cell Line Authentication Service (ATCC) using Short Tandem Repeat (STR) analysis as described in 2012 in the ANSI Standard (ASN‐0002) Authentication of Human Cell Lines [33]. We regularly checked all cell lines for mycoplasma contamination. For spheroids formation, cells after transfection (5 × 10^3^) were seeded in 200 μL of complete growth medium in round bottom, ultra‐low attachment 96‐well plates (#7007; Corning, NY, USA). Spheroids were grown for 10 days. The spheroid size was monitored using live phase‐contrast microscopy (Leica, Wetzlar, Germany).

### 2.9 Transfections

MiRNA mimics were purchased from Sigma‐Aldrich: MISSION miRNA Negative Control 1 (HMC0002) and MISSION microRNA Mimic, hsa‐miR‐200b‐3p (#HM0352). The sense sequence of hsa‐miR‐200b‐3p edited mimics was [AmC6] UCAUCAUUACCAGGCAGCAUUUAdTdT, and the antisense sequence was UAAUGCUGCCUGGUAAUGAUGAdTdT. Silencer® siRNA against ADAR1 (#4390824) and siRNA negative control (#4390843) were purchased from ThermoFisher (USA). The sequence of siRNA for ADAR1 was 5'‐GAGAUUCUCUCAGCCUAAtt. The primer sequences with Assay IDs are assembled in Table S1. According to the manufacturer's instructions, cells were transfected with 50 nm of the indicated miRNA mimics or siRNAs using the DharmaFECT 1 Transfection Reagent (Horizon Discovery, Cambridge, UK) for 24 h. Then, the mixture was removed, and all experiments were conducted with fresh medium, including cell viability assay, colony formation, spheroids formation and scratch assay.

### 2.10 Cell viability assay

Cell viability was measured using the Cell Proliferation Kit I (MTT) (Roche), utilising the ability of viable, metabolically active cells to convert yellow tetrazolium MTT salt (3‐(4,5‐diethylthiazol‐2‐yl)‐2,5‐diphenyltetrazolium bromide) to purple formazan crystals. Cells (2 × 10^3^) were seeded in 96‐well plates, left to attach overnight and treated with 8‐azaadenosine (Tocris Bioscience, Bristol, UK) for 72 h. After exposure, 5 mg·mL^−1^ of the MTT in phosphate‐buffered saline (PBS) was added into each well, and cells were incubated for 4 h at 37 °C. Next, 100 μL of stabilisation buffer (10% SDS in 0.01 M HCl) was added into each well for overnight incubation, followed by absorbance measurement at 540 nm.

### 2.11 Colony survival assay

Cell proliferation was determined by the colony formation assay. Briefly, transfected cells were seeded into 6‐well plates (1 × 10^3^) and cultured for 7 days to form colonies. Finally, the colonies were stained with 0.5% (w/v) crystal violet solution in 50% methanol, photographed, and counted using the ImageJ tool for statistical analysis.

### 2.12 Scratch assay

Cells were transfected with miRNA mimics or siRNA and seeded in 24‐well plates (2 × 10^5^). Twenty‐four hours after transfection, the transfection medium was removed, and the cells grew to 90% confluence. The cell monolayer was scraped in a straight line with a p200 pipette tip to create a scratch. Floating cells and debris were washed with sterile PBS, and the medium was replaced. Plates were subsequently imaged using a live phase‐contrast microscope (Leica) after 0, 12 and 24 h. The distance of a scratch closure was determined using the ImageJ tool. The displayed scratch‐wound healing represents the average from three independent experiments.

### 2.13 Immunohistochemistry

Tissue samples were fixed in 4% paraformaldehyde for 24 h and then paraffin‐embedded using the Excelsior AS tissue processor (Thermo Fisher Scientific). To identify tissue characteristics and structures, haematoxylin and eosin (H&E) staining was performed according to the standard protocol. To evaluate the protein expression and localisation, the IHC was used as follows: 3‐μm microtome sections were placed on glass slides and incubated in PTLink (Tris/EDTA Buffer, pH = 9.0; Agilent Technologies) to retrieve the epitopes that had been masked by fixation. Before a one‐hour incubation in a humid chamber at room temperature with polyclonal rabbit MXI1 antibody (#PA5‐68756; Thermo Fisher Scientific), endogenous peroxidase activity was reduced. EnVision FLEX, High pH (Dako Omnis; Agilent Technologies) visualisation system was used to detect the mentioned proteins. Finally, the slides were counterstained with haematoxylin. The slides were visualised using a digital slide scanner (3DHISTECH PANNORAMIC 250 Flash III DX).

### 2.14 Statistical analyses

Statistical analyses were performed using the GraphPad Prism 9 (v.9.3.1) software. The Mann–Whitney *U* test was used to investigate the age of the patients in the HGSOC patients' group and the healthy group and for editing‐level comparisons between matched tumours and normal samples. Patient survival analysis was performed using the Kaplan–Meier method. All *in vitro* experiment results were compared using ANOVA and a Dunnett test for each comparison. A *P* value < 0.05 was considered significant (*P < 0.05; **P < 0.01; ***P < 0.001; ****P < 0.0001). Correlation analysis was performed using the Pearson correlation coefficient. *P* values < 0.05 were considered significant. Gene Set Enrichment Analysis (GSEA) and enrichment analysis were performed using the WebGestalt R package [34] with a ranked list of all the genes based on the log_10_ (FDR‐corrected *P* value) and a change as up‐ or downregulated. Volcano plots were created in the R environment.

## 3 Results

### 3.1 Identification of A‐to‐I editing hotspots in miRNAs of HGSOC patients

**Fig. 1:**
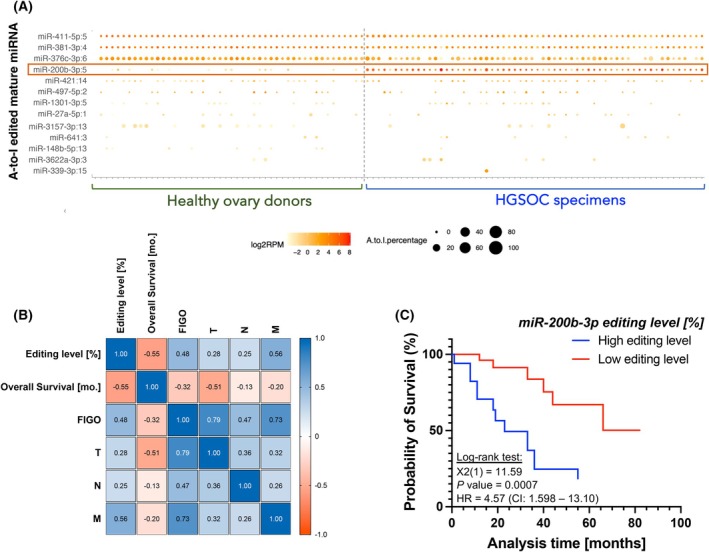
Clinically relevant patterns of A‐to‐I editing in HGSOC patient samples. (A) Heat map of miRNA editing hotspots. Edited miRNA expression amounts (log_2_RPM) are in colour; editing frequencies (% of samples with editing signals) are indicated by circle size. The red box highlights miR‐200b‐3p:5, which shows the most significantly altered miRNA A‐to‐I editing levels in HGSOC specimens compared to healthy ovaries. (B) Pearson correlation results of the miRNA's editing level [%] and clinical features: overall survival, FIGO stage, and tumour‐node‐metastasis (TNM) stage. The colour scale (blue to red) indicates correlation. The blue colour indicates a positive correlation, and red indicates the opposite. (C) Kaplan–Meier plot of patients grouped by editing level of miR‐200b‐3p (high expression > 50% of the median; low expression < 50% of the median).

To comprehensively characterise the profile of miRNA A‐to‐I editing events in 60 HGSOC tissues and 48 control ovarian tissues, we used the miRge 3.0 package [23]. We identified 13 unique A‐to‐I editing hotspots (Fig. 1A). The mean level of A‐to‐I editing at the 13 sites ranged from 0.01% to 11.54% across tested individuals. Nine of the 13 A‐to‐I edited sites were in miRNA seed regions (Positions 1–5). Position 5 of miR‐200b‐3p was significantly more frequently edited in HGSOC specimens than in healthy ovaries (*P* = 0.0015) (Table S2). miR‐200b‐3p plays an essential role in cancer metastasis by inhibiting epithelial–mesenchymal transition (EMT) [13]. Consistent with its established role as a tumour suppressor, high wild‐type miR‐200b‐3p expression is associated with better clinical outcomes across kidney, lung, and stomach cancer [7]. To further pinpoint the clinical role of the miR‐200b‐3p editing events in HGSOC, Pearson's correlation coefficients were used to assess the correlation between A‐to‐I editing of miR‐200b‐3p at Position 5 and the following clinical characteristics: overall survival [mo.], FIGO stage, and tumour‐node‐metastasis (TNM) staging. We observed a positive correlation between the % of editing level of miR‐200b‐3p in HGSOC patients and the FIGO stage, as well as the occurrence of metastasis, with a correlation coefficient (*r*) of 0.48 and 0.56, respectively (*P* = 0.0031 and *P* = 0.00034). In contrast, the miR‐200b‐3p editing level was negatively correlated with the overall survival (*r* = −0.56, *P* = 0.00024) (Fig. 1B). In addition, as shown by the Kaplan–Meier survival analysis, the higher miR‐200b‐3p editing level in HGSOC patients was associated with worse patient survival (Fig. 1C). These results suggest that miR‐200b‐3p following A‐to‐I editing (ED‐miR‐200b‐3p) may lose its tumour suppressor function and facilitate HGSOC progression.

### 3.2 Functional effects of RNA editing in miR‐200b‐3p on HGSOC cells proliferation, migration and 3D growth

**Fig. 2:**
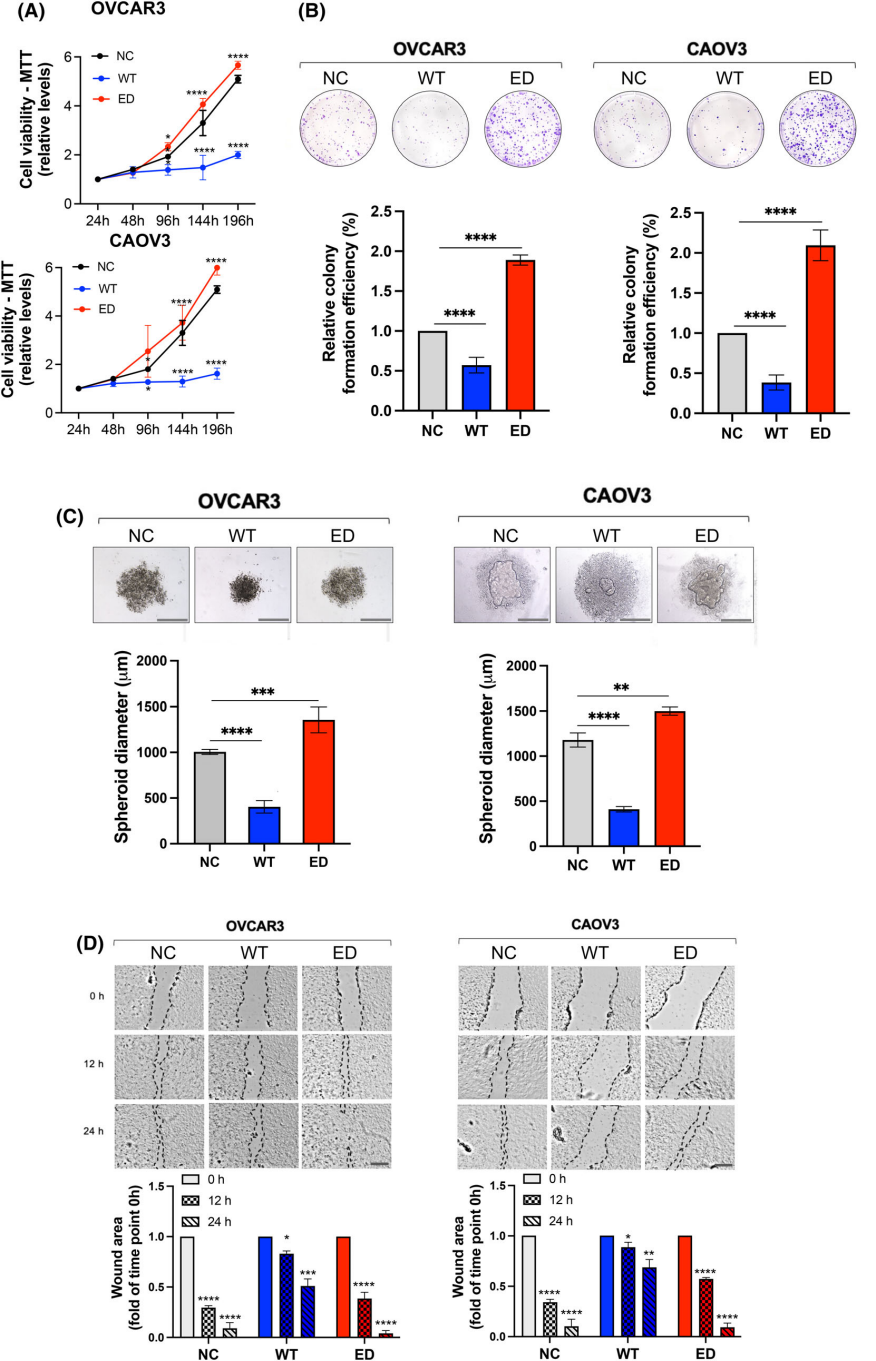
Editing of miR‐200b‐3p induces cell proliferation, colony formation, migration, and 3D growth. (A) MTT assay at the indicated time points. Results are mean ± SD, *n* = 3. Significance: Two‐way ANOVA; *P < 0.05, ****P < 0.0001. (B) Upper panel: Representative images of crystal violet‐stained colonies. Bottom panel: Quantitative analysis of colony formation assay. Results are mean ± SD, *n* = 6. Significance: Student's *t*‐test analysis; ****P < 0.0001. (C) Representative images of 3D spheroids culture and changes in spheroid diameter. Scale bar = 500 μm. Results are mean ± SD, *n* = 3. Significance: Student's *t*‐test analysis; **P < 0.01, ***P < 0.001, ****P < 0.0001. (D) Representative bright‐field images show that the acceleration of gap closure varied between cells transfected with WT‐miR‐200b‐3p, ED‐miR‐200b‐3p mimics or negative control (NC) in OVCAR3 and CAOV3 cells. Wound healing expressed as the remaining area uncovered by the cells was calculated using the ImageJ software. Scale bar = 100 μm. Results are mean ± SD, *n* = 3. Significance: Student's *t*‐test analysis; *P < 0.05, **P < 0.01, ***P < 0.001, ****P < 0.0001.

To further investigate the functional consequences of miR‐200b‐3p editing events, we used chemically synthesised WT and ED‐miR‐200b‐3p RNA mimics and examined the effect of miRNA overexpression on proliferation, migration and 3D growth in high‐grade serous ovarian cancer, OVCAR3 and CAOV3 cell lines. Cells transfected with ED‐miR‐200b‐3p mimic showed significantly increased viability and colony formation potential compared with control cells (Fig. 2A, B, Fig. S1A,B). In contrast, transfection of WT‐miR‐200b‐3p mimics dramatically decreased cell proliferation and colony formation, corresponding to WT‐miR‐200b‐3p function as a tumour suppressor (Fig. 2A, B). Next, we confirmed these observations using three‐dimensional (3D) OVCAR and CAOV3 spheroids that better recapitulate tumour physiology than regular 2D cell culture. WT miR‐200b‐3p mimics effectively blocked the growth of spheroids derived from both cell lines, while ED‐miR‐200b‐3p mimic markedly accelerated spheroid expansion and size (Fig. 2C). Next, we performed a scratch‐wound healing assay to determine the effect of miR‐200b‐3p editing on cancer cell migration. In both OVCAR3 and CAOV3 cell lines, transfection of the WT‐miR‐200b‐3p mimic resulted in scratch closure at a much slower rate than in the negative control (NC). In contrast, overexpression of ED‐miR‐200b‐3p mimic significantly promoted the motility of OVCAR3 and CAOV3 cells, leading to rapid wound healing (Fig. 2D). These results suggest that A‐to‐I edited miR‐200b‐3p may act as an oncomiR, and the increased RNA editing level in ovarian cancer cells may be relevant to tumour progression.

### 3.3 *ADAR1* silencing abolishes the effect of miR‐200b‐3p edition level

**Fig. 3:**
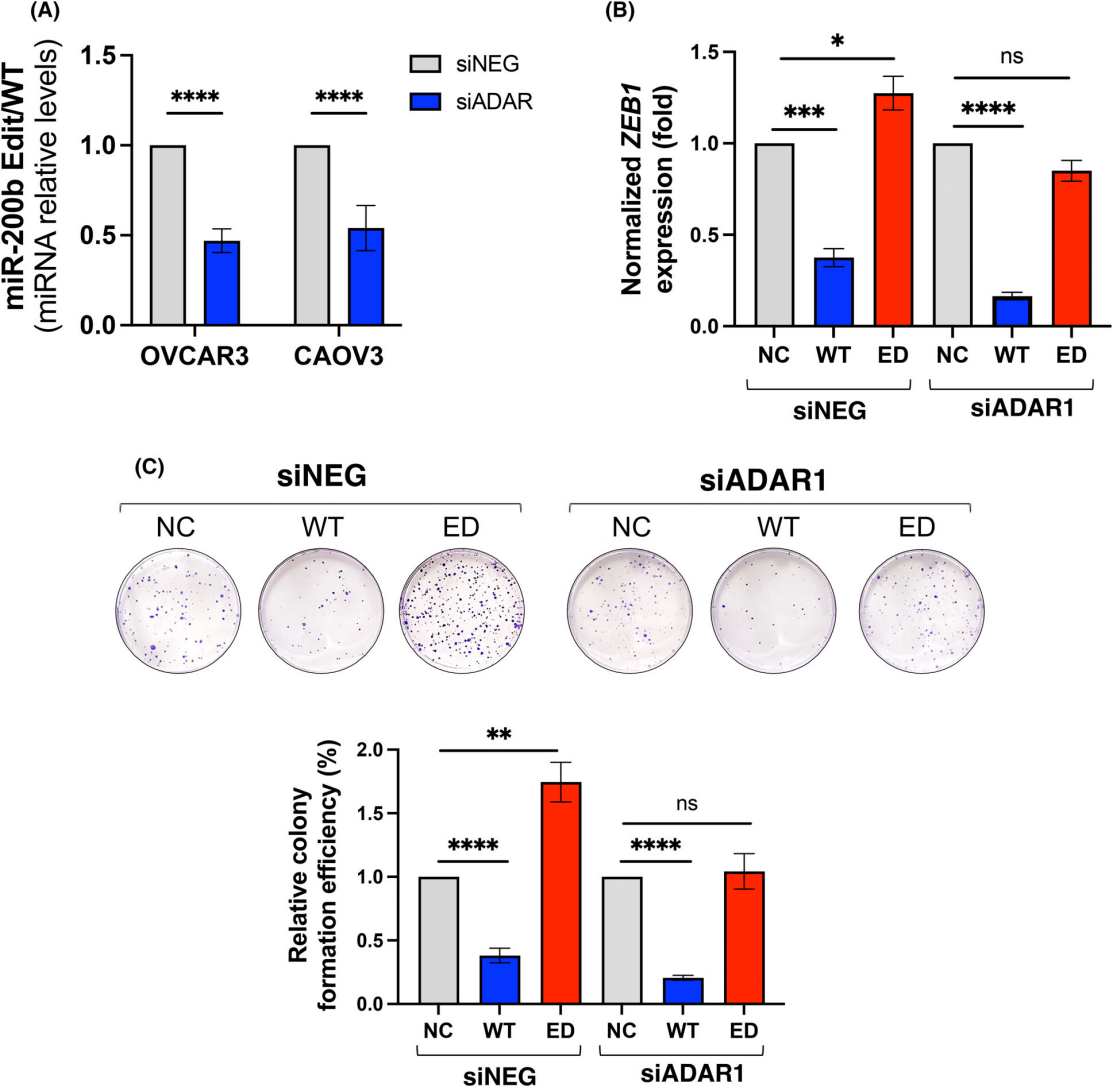
Effect of ADAR1 silencing on miR‐200b‐3p editing level and ZEB1 expression. Effect of ADAR1 silencing on editing levels and ZEB1 expression. (A) miR‐200b Edit/WT miRNA relative levels in OVCAR3 and CAOV3 cells transfected with 50 nm siADAR1 and siNEG (siControl) for 24 h. Results are mean ± SD, *n* = 3. Significance: Student's *t*‐test analysis; ****P < 0.0001. (B) Quantitative reverse transcriptase PCR (RT‐qPCR) of *ZEB1* gene upon 24 h cotransfection with 50 nm siNEG (negative control), siADAR1, ED‐miR‐200b‐3p, WT‐miR‐200b‐3p mimics, or negative control (NC) in OVCAR3 and CAOV3 cells. Results are mean ± SD, *n* = 3. Significance: Student's *t*‐test analysis; *P < 0.05, ***P < 0.001, ****P < 0.0001. (C) Upper panel: Representative images of crystal violet‐stained colonies. Bottom panel: Quantitative analysis of colony formation assay of cotransfected cells. Results are mean ± SD, *n* = 6. Significance: Student's *t*‐test analysis; **P < 0.01, ****P < 0.0001.

It has been reported that ADAR enzymes, specifically ADAR1, catalyse A‐to‐I miRNA editing. ADAR1 is believed to play a critical oncogenic role. Increased *ADAR1* expression and/or activity has been implicated in many cancers, and its overexpression is associated with an unfavourable prognosis [35, 36]. To investigate whether abnormal expression of *ADAR1* in ovarian cancer cell lines affects miR‐200b‐3p editing, we analysed the miR‐200b edited/WT ratio in ADAR1‐silenced OVCAR3 and CAOV3 cells. We found that the ED‐/WT‐miR‐200b‐3p ratio decreased, indicating a suppressed A‐to‐I editing of miR‐200b‐3p (Fig. 3A, Fig. S1C,D). Wild‐type miR‐200b‐3p has been described as a tumour suppressor miRNA that disrupts the EMT program. One of the main targets for wild‐type miR‐200b‐3p is *ZEB1* (zinc finger E‐box‐binding homeobox 1), a critical activator of EMT in cancer [37]. We found that silencing *ADAR1* expression resulted in more effective inhibition of *ZEB1* expression levels and reduced clonogenic potential in WT‐miR‐200b‐3p transfected cells (Fig. 3B, C). However, transfection of the ED‐miR‐200b‐3p mimic of ADAR1‐silenced cells does not entirely reverse the silencing effect. This observation is supported by the fact that ADAR1 plays a significantly broader role in cancer cell biology than only the function as a catalytic mediator of A‐to‐I editing in miR‐200b‐3p.

Next, we studied the functional effects of *ADAR1* knockdown on cell growth and observed that *ADAR1* silencing profoundly suppressed cell viability and clonogenic potential (Fig. S2A,B). In addition, ADAR1‐knocked down ovarian cancer cells formed smaller and more compact spheroids than control cells, NC (Fig. S2C). Furthermore, ADAR1 downregulation completely prevented scratch closure in OVCAR3 cells and significantly reduced wound healing in the CAOV3 cell line (Fig. S2D). In addition, pharmacological inhibition of ADAR1 with 8‐azaadenosine (8‐aza) showed a strong dose‐dependent antiproliferative effect, as measured by MTT assay (Fig. S2E).

### 3.4 Effects of miR‐200b‐3p editing on transcriptome profile of HGSOC cells

**Fig. 4:**
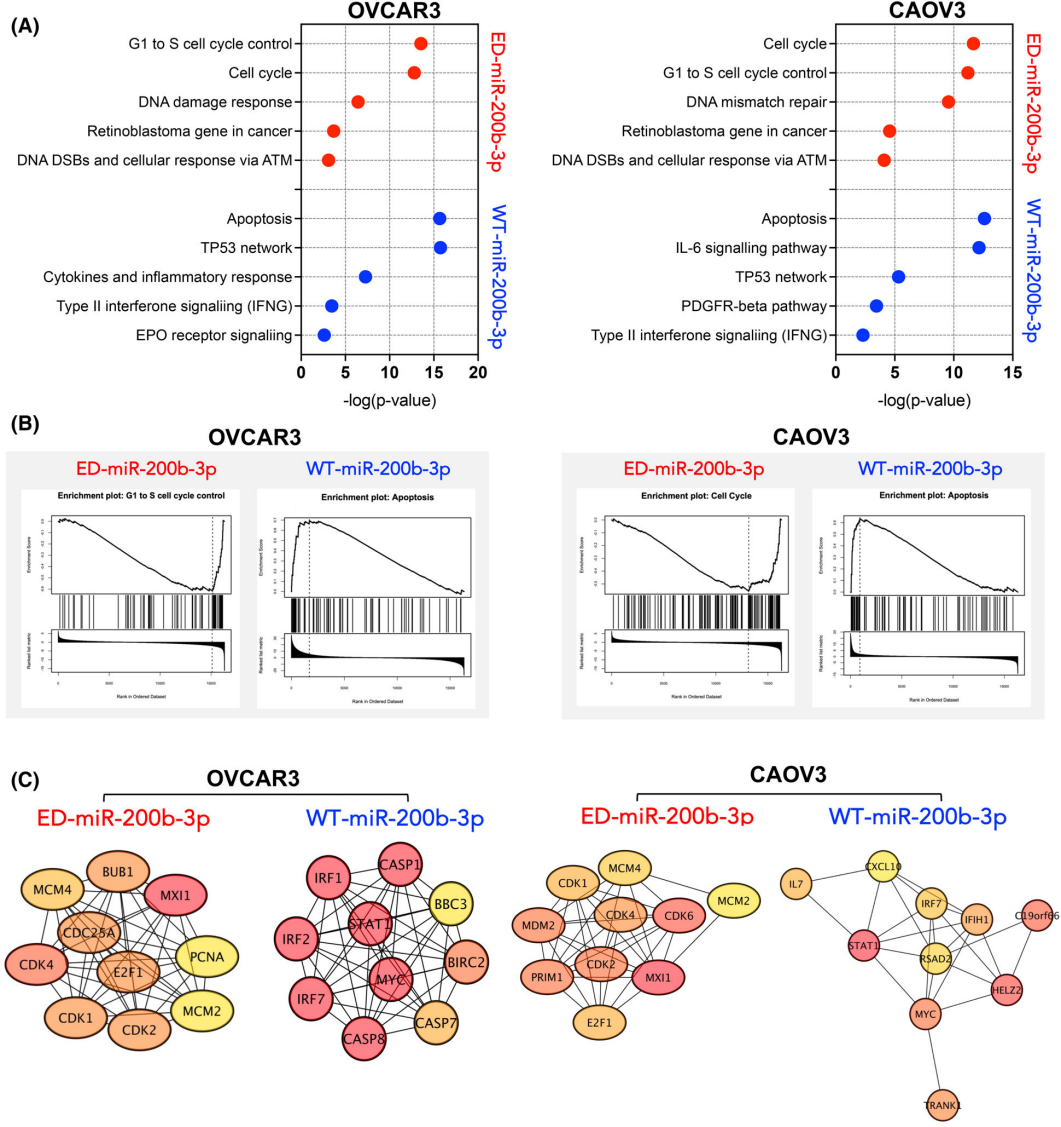
miR‐200b‐3 editing effects on transcriptome profile of HGSOC cells. (A) WikiPathways analysis for cancer‐related pathways enrichment analysis of DEGs in OVCAR3 and CAOV3 cells transfected with 50 nm ED‐miR‐200b‐3p and WT‐miR‐200b‐3p mimics for 24 h. (B) Gene set enrichment analysis (GSEA) in DEGs in OVCAR3 and CAOV3 cells upon transfection of 50 nm ED‐miR‐200b‐3p and WT‐miR‐200b‐3p for 24 h. (C) The network of the top 10 hub genes in OVCAR3 and CAOV3 cells transfected with 50 nm ED‐miR‐200b‐3p and WT‐miR‐200b‐3p mimics for 24 h.

Given the potential effect of A‐to‐I editing events on global differences in the transcriptome, we also performed RNA‐Seq analysis in OVCAR3 and CAOV3 after 24‐h transfection with WT‐ and ED‐miR‐200b‐3p mimics. The selection criteria for differentially expressed genes (DEGs) analysis included the threshold of FDR ≤ 0.05 and |log_2_FC| ≥ 1. Volcano plots were plotted to illustrate the distribution of each gene according to the log_2_FC and FDR (Fig. S3). Overexpression of the WT‐miR‐200b‐3p mimic resulted in 912 and 263 DEGs in OVCAR3 and CAOV3, whereas the ED‐miR‐200b‐3p mimic led to 273 and 121 DEGs in OVCAR3 and CAOV3 cells, respectively. Next, we constructed a protein–protein interactions (PPI) network of the DEGs in transfected cells using the STRING database in Cytoscape version 3.10.1 (https://cytoscape.org). The top 10 ranked nodes were selected using the maximum clique centrality (MCC) method in the *cytoHubba* plugin [38] in Cytoscape (Fig. 4C). These 10 genes were used as hub genes and were shown with red (high ranking) and yellow nodes low ranking based on the ranking score. The top 10 hub genes in OVCAR3 cells transfected with the ED‐miR‐200b‐3p mimic comprised *MXI1*, *CDK4*, *CDC25A*, *E2F1*, *BUB1*, *CDK2*, *CDK1*, *MCM4*, *MCM2* and *PCNA*. In CAOV3 cells transfected with the ED‐miR‐200b‐3p mimic, we identified *MXI1*, *CDK6*, *PRIM1*, *CDK2*, *MDM2*, *CDK4*, *E2F1*, *CDK1*, *MCM4* and *MCM2* as putative hub genes. In OVCAR3 cells transfected with the WT‐miR‐200b‐3p mimic, the highly connected hub genes were *MYC*, *BIRC2*, *IRF7*, *IRF1*, *CASP7*, *CASP1*, *IRF2*, *CASP8*, *BBC3* and *TNFSF10*. In the CAOV3 cells transfected with the WT‐miR‐200b‐3p mimic, the top 10 high‐degree genes were *STAT1*, *HELZ2*, *C19orf66*, *MYC*, *TRANK1*, *IFH1*, *IL7*, *IRF7*, *RSAD2* and *CXCL10*. To better understand the biological function of identified DEGs, WikiPathways analysis for cancer‐related pathways was performed. In ED‐miR‐200b‐3p mimic transfected cells, dysregulated pathways were mainly associated with the cell cycle and DNA damage response (Fig. 4A). Furthermore, gene set enrichment analysis (GSEA) [39] and key hub genes showed that edited miR‐200b‐3p significantly inhibited G1 to S cell cycle control (NES = −1.89, FDR = 0.01) in OVCAR3, and cell cycle (NES = −1.71, FDR = 0.01) in CAOV3 (Fig. 4B, C). In contrast, as shown by WikiPathways analysis, the WT‐miR‐200b‐3p mimic in OVCAR3 and CAOV3 induced significant changes in the expression of genes involved in p53‐dependent apoptosis pathways (Fig. 4A, B, C). Correspondingly, GSEA indicated activation of the apoptotic signalling pathway in OVCAR3 (NES = 2.41, FDR < 2.2 × 10^−16^) and CAOV3 (NES = 2.04, FDR < 2.2 × 10^−16^) upon WT miR‐200b‐3p mimic treatment consistent with tumour suppressor function of non‐edited miR‐200b‐3p. In support of these observations, analysis of caspase 3/7 activity indicated that miR‐200b‐3p editing may protect ovarian cancer cells from undergoing apoptosis. In cells transfected with the WT‐miR‐200b‐3p mimic, caspase 3/7 activity increased substantially compared to control cells (Fig. S4A). Furthermore, ADAR1 silencing prevented the A‐to‐I editing and resulted in increased activation of caspase 3/7 (Fig. S4B). In contrast, the ED‐miR‐200b‐3p mimic decreased the caspase 3/7 activity (Fig. S4A). Collectively, these results suggest that miR‐200b‐3p editing events may influence ovarian cancer cell survival by promoting or inhibiting apoptosis.

### 3.5 A‐to‐I editing in miR‐200b‐3p changes its target genes in HGSOC cells

**Fig. 5:**
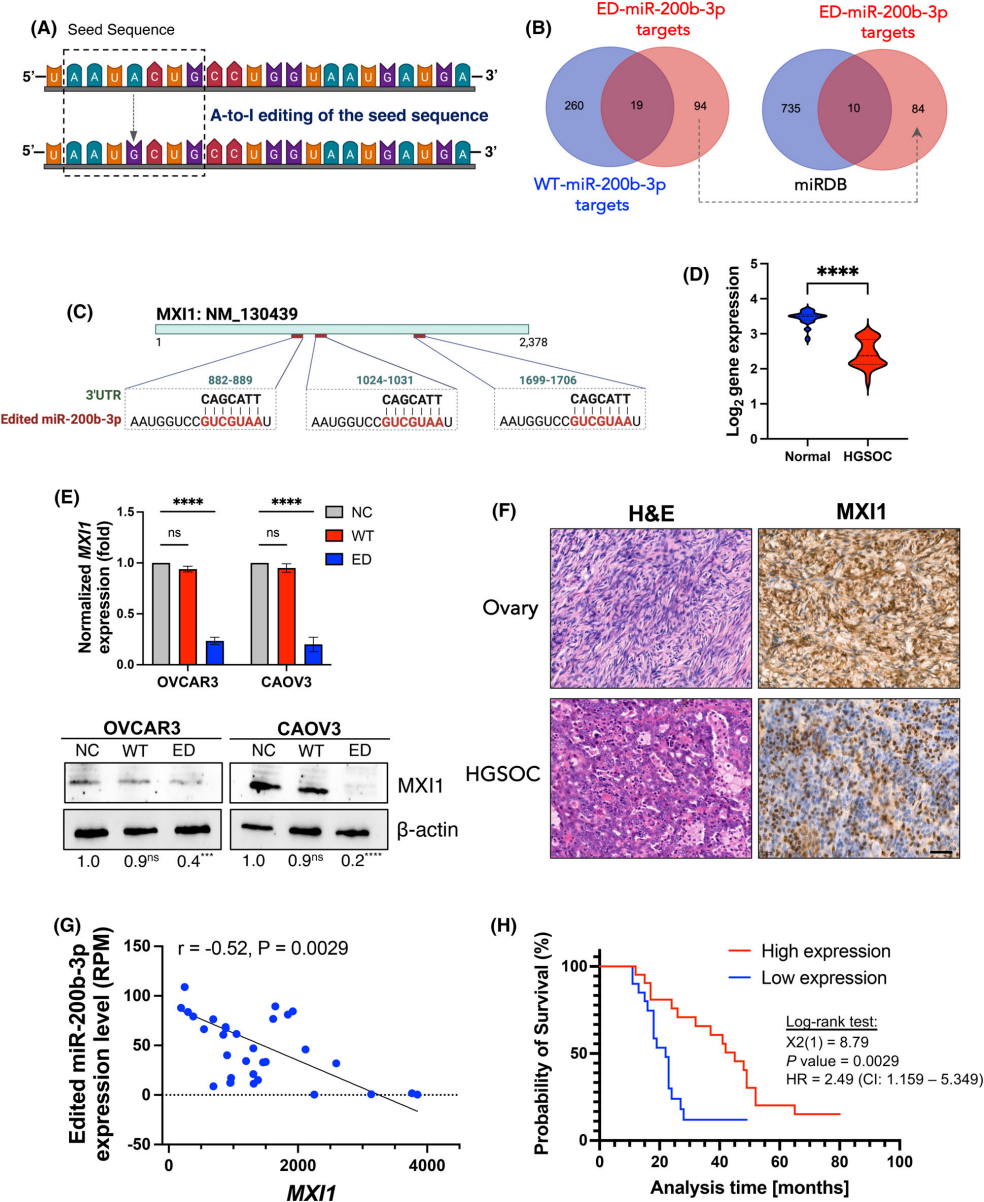
A‐to‐I editing in miR‐200b‐3p redirects the target genes in ovarian cancer cells. (A) Schematic of the A‐to‐I edited site in the seed region of mature miR‐200b‐3p in HGSOC patients. (B) Venn diagrams represent the identification of WT‐miR‐200b‐3p and ED‐miR‐200b‐3p target genes using RNA‐Seq data and miRDB. (C) 3′UTR representations of edited miR‐200b‐3p target gene *MXI1*. (D) Expression level obtained by RNA‐Seq of the MXI1 gene in HGSOC tissues compared to control ovarian tissues. Significance: Student's *t*‐test analysis; ****P < 0.0001. (E) Quantitative reverse transcriptase PCR (RT‐qPCR) of the *MXI1* gene and protein level of MXI1 upon 24‐h transfection with 50 nm ED‐miR‐200b‐3p, WT‐miR‐200b‐3p mimics, or negative control (NC) in OVCAR3 and CAOV3 cells. Significance: Student's *t*‐test analysis; ****P < 0.0001. Results are mean ± SD, *n* = 3. The protein level was analysed using western blotting. Bands were analysed by densitometry using ImageJ software and normalised to β‐Actin. (F) Representative H&E staining of HGSOC and control ovarian tissues and IHC analysis of MXI1 protein expression in HGSOC and control ovarian tissues. Scale bar = 50 μm. (G) Pearson correlation of miR‐200b‐3p editing level (RPM) and *MXI1* expression level in HGSOC patients. (H) Kaplan–Meier plot of patients grouped by *MXI1* expression level (high expression > 50% of the median; low expression < 50% of the median).

A‐to‐I editing in miR‐200b‐3p may change its target because the RNA editing occurred in the seed region (nucleotide position 2–8 from the 5′ end of the miRNA) (Fig. 5A). Even though base pairing between miRNA and its target mRNA is not ideally matched, the seed region has to be perfectly complementary to the miRNA sites in the mRNA 3'UTR [40, 41]. Therefore, we performed gene set enrichment to test whether the potential WT‐miR‐200b‐3p or ED‐miR‐200b‐3p target genes were enriched in downregulated DEGs. Indeed, we found that miRDB‐predicted (MicroRNA Target Prediction Database; https://mirdb.org) WT‐miR‐200b‐3p putative targets were significantly enriched in downregulated DEGs in OVCAR3 (NES = −1.51, FDR < 2.2 × 10^−16^) and CAOV3 (NES = −1.55, FDR < 2.2 × 10^−16^) transfected with WT‐miRNA‐200b‐3p mimic. On the other hand, ED‐miR‐200b‐3p targets were significantly enriched in downregulated mRNAs in OVCAR3 (NES = −2.34, FDR < 2.2 × 10^−16^) and CAOV3 (NES = −1.95, FDR < 2.2 × 10^−16^) transfected with ED‐miR‐200b‐3p mimic (Fig. S5). According to this global analysis, editing of miR‐200b‐3p at the single‐nucleotide level redirects the miRNA to inhibit a new set of target genes by altering the seed sequence complementarity.

**Fig. 6:**
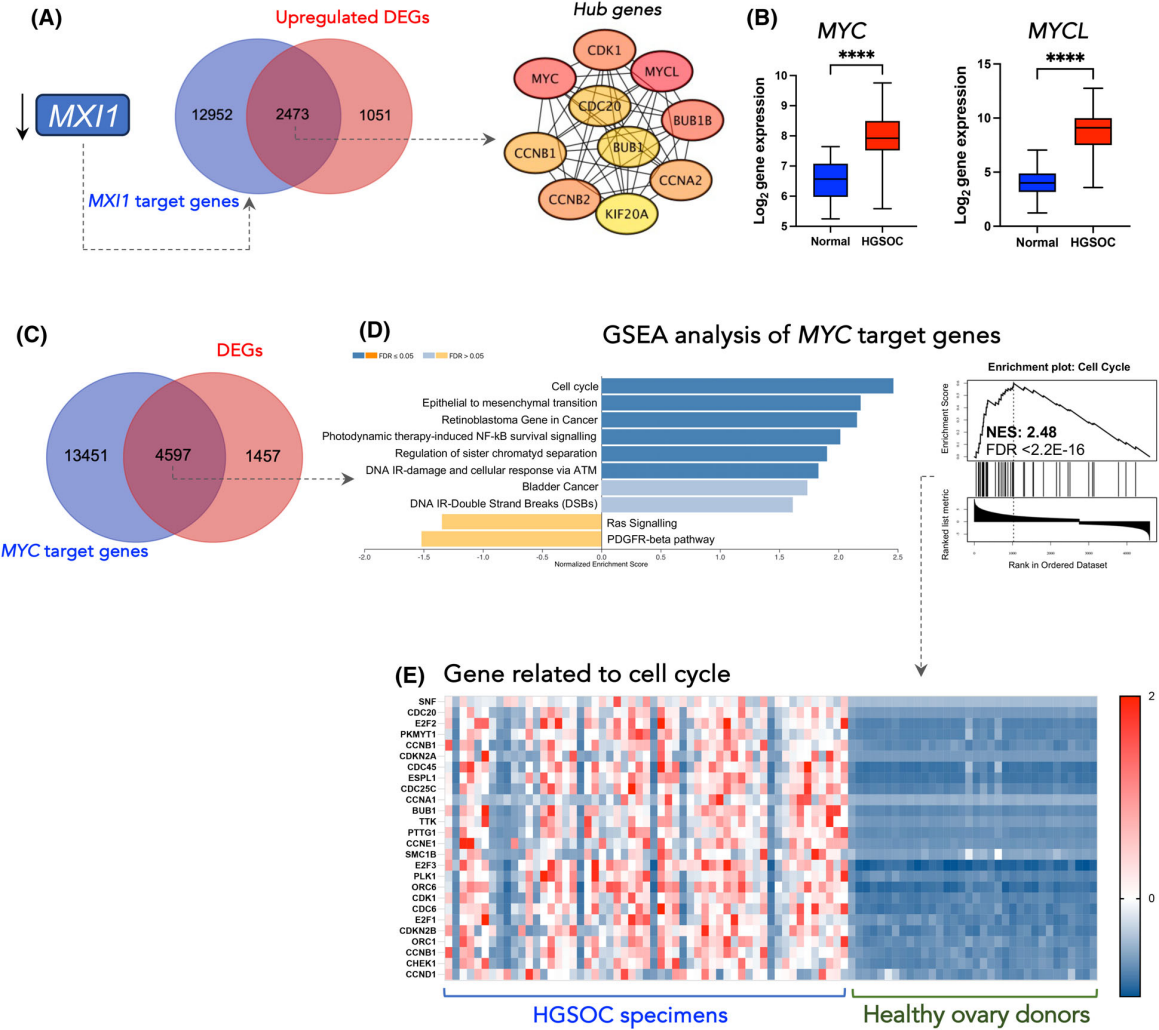
Downregulation of *MXI1* effects of its target set. (A) Venn diagram showing the number of overlapped MXI1 targets between DEGs and putative targets based on miRDB, as well as the network of the top 10 hub genes from overlapped MXI1 target genes; miRDB–microRNA Target Prediction Database. (B) Expression level obtained by RNA‐Seq of *MYC* and *MYCL* gene in HGSOC tissues compared to control ovarian tissues. Significance: Student's *t*‐test analysis; ****P < 0.0001. Results are mean ± SD. (C) Venn diagram showing the number of MYC targets between DEGs and putative targets based on miRDB. (D) GSEA of MYC target genes. (E) Heat map of DEGs related to cell cycle through GSEA.

To identify key targets of edited miR‐200b‐3p, we listed identified genes from the RNA‐Seq experiment that comply with the following criteria: genes that (a) were only significantly downregulated (FDR ≤ 0.05 and |log_2_FC| ≥ 1) in cells upon transfection of ED‐miR‐200b‐3p mimic (relative to the control mimics); (b) were overlapped with the putative targets list for ED‐miR‐200b‐3p in the miRDB database; (c) had at least one binding site for ED‐miR‐200b‐3p but no binding site for WT‐miR‐200b‐3p in the 3'‐UTR region. This classification yielded 10 candidate targets of ED‐miR‐200b‐3p including *MXI1*, *TSHZ3*, *F3*, *OTX1*, *RC3H2*, *TRAM1*, *UBXN2B*, *PAQR3*, *DIXDC1* and *RAB5C*. All selected targets had a prediction score in the range of 50–100 as assigned by the miRDB database, with a higher score representing higher statistical confidence in the prediction result (Fig. 5B and Table S3). The top candidate, MAX interactor 1 (*MXI1*) (FDR = 5.74 × 10^−19^), has three binding sites in its 3'‐UTR regions for the ED‐miR‐200b‐3p (Fig. 5C and Fig. S6). Importantly, MXI1 is a transcriptional factor that antagonises the oncogenic activity of c‐MYC to suppress tumour development [42]. To support miRNA target prediction, we found that overexpression of ED‐miR‐200b‐3p mimic significantly reduced the expression and protein level of MXI1 in OVCAR3 and CAOV3 cell lines (Fig. 5E). Similarly, MXI1 was downregulated at the mRNA and protein levels in specimens from HGSOC patients (Fig. 5D, F). The expression of *MXI1* in HGSOC samples was moderately negatively correlated with the level of edited miR‐200b‐3p (*r* = −0.52) with a significant statistical value of *P* = 0.0029 (Fig. 5G). Kaplan–Meier survival analysis showed that HGSOC patients with low *MXI1* expression (low expression < 50% of the median) had a dismal overall survival rate (Fig. 5H). As *MXI1* expression was downregulated in HGSOC patients and MXI1 is known as an oncogene suppressor, next we determined whether its known target genes were enriched among upregulated DEGs. The 2473 targets overlapped between 3524 upregulated DEGs and 15 425 target genes via the ENCODE Transcription Factor Targets Dataset [43, 44] (https://maayanlab.cloud/Harmonizome/dataset/ENCODE+Transcription+Factor+Targets) (Fig. 6A). Next, based on the constructed protein–protein interactions (PPI) network of the overlapped DEGs, we identified the top 10 hub genes, which included *MYCL*, *MYC*, *BUB1B*, *CDK1*, *CCNB2*, *CCNA2*, *CCNB1*, *CDC20* and *BUB1*. MYC oncogene family consists of three interrelated nuclear phosphoproteins (c‐Myc, L‐myc‐1 and N‐Myc) that play a crucial role in various cellular pathways associated with the pathogenesis of cancer and are essential for the survival of cancer cells [45]. Notably, the overexpression of *MYC* and *MYCL* has been revealed in multiple types of cancer [42]. To support MXI1 target predictions we determined the expression of *MYC* and *MYCL* in HGSOC tissue. Consistent with decreased levels of MXI1, both genes were overexpressed in HGSOC patients compared to normal specimens (Fig. 6B). Next, we identified MYC targets by comparing 6054 DEGs and 18 048 targets from the ENCODE Transcription Factor Targets Dataset and performed GSEA based on obtained 4597 overlapped genes (Fig. 6C). MYC targets were the most involved in the activation of the cell cycle (NES = 2.48, FDR < 2.2 × 10^−16^) but also in epithelial to mesenchymal transition (NES = 2.45, FDR < 2.2 × 10^−16^) (Fig. 6D). Consistent with computational GSEA, genes associated with the cell cycle pathway (*SNF*, *CDC20*, *E2F2*, *PKMYT1*, *CCNB1*, *CDKN2A*, *CDC45*, *ESPL1*, *CDC25C*, *CCNA1*, *BUB1*, *TTK*, *PTTG1*, *CCNE1*, *SMC1B*, *E2F3*, *PLK1*, *ORC6*, *CDK1*, *CDC6*, *E2F1*, *CDKN2B*, *ORC1*, *CCNB1*, *CHEK1* and *CCND1*) were overexpressed in HGSOC but not healthy ovary tissues (Fig. 6E). Altogether, these results suggested that A‐to‐I editing of miR‐200b‐3p may result in decreased expression of *MXI1* leading to upregulation of *MYC* and *MYCL* expression, contributing to poor prognosis in HGSOC patients.

## 4 Discussion

Ovarian cancer remains the most lethal among female cancers. The average survival time for high‐grade serous ovarian cancer (HGSOC) is around 3.4 years after diagnosis [46]. Treatment options are limited, especially for patients with relapse who are resistant to standard treatment. Thus, the search for new mechanisms of tumorigenesis is crucial to developing treatment strategies for HGSOC therapy. A‐to‐I editing is one of the most commonly detected post‐transcriptional RNA modifications. Interestingly, RNA editing has been observed in both coding and noncoding genes, including miRNAs. Editing events, particularly in the seed regions of mature miRNA sequences, can significantly alter the spectrum of miRNA targets, also known as the ‘targetome’, and subsequently modify their function [47]. Therefore, in the case of cancer, the editing process may transform specific miRNAs that suppress tumours into miRNAs that promote oncogenesis [48]. Here, we focused on A‐to‐I editing events in miRNAs in HGSOC patients. We functionally characterised an A‐to‐I editing at position 5 of miR‐200b‐3p, which was the most significantly modified site in tested HGSOC tissue compared with normal ovaries. The same A‐to‐I edited hot spot in miR‐200b‐3p has also been reported in various tumours, including breast, colon, head and neck, kidney, lung, stomach, thyroid and uterus [7]. In our study, we observed a positive correlation between the level of miR‐200b‐3p editing and the FIGO stage as well as the occurrence of metastasis and a negative correlation with the HGSOC patient's overall survival. Furthermore, a higher miR‐200b‐3p editing level was associated with a worse patient prognosis. Our results also demonstrated that editing of miR‐200b‐3p at position 5 was sufficient to significantly promote cell proliferation and accelerate migration in HGSOC cell lines (OVCAR3 and CAOV3). Importantly, unedited miR‐200b‐3p suppressed cell viability and motility. Functionally, miR‐200b‐3p acts as a tumour suppressor in various cancers, including gastric cancer [49], hepatocellular carcinoma [50], breast cancer [51], glioma [52], and prostate cancer [53]. miR‐200b‐3p has been previously reported to be also downregulated in most ovarian cancer patients classified with a risk of relapse compared with no‐relapsers [54]. Loss of miR‐200b‐3p upregulates endothelin‐1 receptor (ET_A_R) and ZEB1 axis, which fosters ovarian cancer progression [55].

The enzyme ADAR1 mainly directs A‐to‐I editing in multiple human cancers [56]. Our study shows that the miR‐200b editing level was dependent on the level of *ADAR1* gene expression. We observed that repressed *ADAR1* expression notably suppressed the proliferation, migration, and 3D growth of HGSOC cells. Furthermore, we demonstrated that the treatment of ovarian cells with 8‐azaadenosine, an inhibitor of ADAR1 activity and A‐to‐I editing, reduced cell viability in a time‐ and dose‐dependent manner, suggesting that miRNA editing may represent a promising therapeutic strategy for HGSOC. A similar effect has been previously observed in thyroid cancer [12]. Additionally, ADAR1 has been suggested as a novel target for immunotherapy in cancer. A recent study demonstrated that silencing ADAR1 could increase the susceptibility of tumour cells to immunotherapy [57, 58]. These and our observations raise the need for the development of new molecules targeting ADAR1 activity.

Given that miRNA function strongly depends on sequence complementarity with target mRNA, each of the A‐to‐I editing events identified within the seed sequence results in a change in the miRNA targetome and consequently modifies its primary function [9]. Thus, we also assessed the shift in the targetomes of canonical and edited miR‐200b‐3p. First, we identified high‐confidence target candidates of WT‐ and ED‐miR‐200b‐3p by integrating differential expression of genes and binding motif information and found that only 19 genes overlapped between these two target gene sets. Among the potential targets of the edited miR‐200b‐3p, we identified the *MXI1* gene, which encodes the MAX interactor 1, as the target with the highest number of binding sites for ED‐miR‐200b‐3p. Importantly, WT‐miR‐200b‐3p did not present any binding sites for *MXI1*, confirming ED‐miR‐200b‐3p target selectivity. Furthermore, *MXI1* also proved to be the top hub gene in the PPI network in tested HGSOC cell lines *in vitro*, with the highest degree of connectivity. The protein encoded by this gene is a member of the mitotic arrest deficient (MAD) family, which inhibits proliferation and induces the accumulation of cells in the G0/G1 cell cycle phase [59]. Tumour suppressor roles of MXI1 have been validated in different types of cancers, including prostate cancer [60], glioblastoma [61] and lung cancer [62]. Importantly, it has also been demonstrated that *MXI1* was significantly downregulated in lung tumours, and the downregulation was associated with poor prognosis. In our study, we also found that MXI1 was downregulated in HGSOC tissues compared to healthy ovaries at the mRNA and protein levels, and this inhibition was negatively correlated with the miR‐200b‐3p editing level. Importantly, MXI1 is a transcriptional repressor of the MYC oncogene. MXI1 specifically binds with MAX to form a sequence‐specific DNA‐binding protein complex that recognises the core sequence 5′‐CAC[GA]TG‐3′. Thus, MXI1 antagonises MYC transcriptional activity by competing for MAX. It has been well documented that MXI1, through negative regulation of MYC, exerts an inhibitory effect on various types of malignancies [63, 64, 65]. Our studies support these findings and demonstrate the upregulation of *MYC* and *MYCL* expression in HGSOC patients. Reduced *MXI1* can no longer sufficiently prevent transcriptional activity of MYC, thus fuelling malignant progression. Indeed, as confirmed by our RNA‐Seq analysis, HGSOC patients had upregulated expression of several MYC targets that drive cell cycle progression. These included cyclin‐dependent kinases (*CDK1*, *CHEK1*, *BUB1*, *PLK1*), cyclins (*CCNA1*, *CCNB1*, *CCND1*, *CCNE1*, *CDC25C*, *CDC6*, *CDC20*, and *CDC45*) and E2F transcription factor (*E2F1*, *E2F2*). These observations are in agreement with well‐documented evidence that the MYC oncogene promotes cell cycling, mainly through the activation of cyclins and CDKs.

It is essential to acknowledge the limitations of the study, which should be transparently articulated. First, the study population size is a significant limitation. However, this is a substantial sample size, particularly when considering the costly and laborious nature of small RNA‐seq analyses. It is imperative to validate the obtained results through further external and independent means in the long‐term follow‐up cohorts. Additionally, some patients were lost to follow up within shorter periods than the study duration due to various reasons. The study population was of European ethnicity, so caution should be taken while extrapolating to other ethnic groups where the survival may differ. It should also be noted that to investigate the translational potential of edited miR‐200b‐3p, it is essential to conduct *in vivo* experiments in multiple mouse models. Further efforts should be made to investigate whether inhibitors of edited miR‐200b‐3p can be combined with other anticancer therapies to enhance therapeutic efficacy.

## 5 Conclusion

Our findings indicate that excessive ADAR1‐dependent editing of canonical miR‐200b‐3p plays a critical role in the pathogenesis and progression of high‐grade serous ovarian cancer. A‐to‐I editing alters the seed sequence complementarity and redirects miR‐200b‐3p function to differentially regulate a novel set of target genes. This functional change had its consequences in acquiring the new ability of ED‐miR‐200b‐3p to block *MXI1*, which reduced expression and was substantially related to poor prognosis in HGSOC patients. Broadly, edited miR‐200b‐3p may represent a novel cancer therapeutic strategy for ovarian cancer patients.

## Conflict of interest

The authors declare no conflict of interest.

## Author contributions

**MN:** Conceptualisation, Methodology, Validation, Formal analysis, Investigation, Writing – Original Draft, Visualisation, Funding acquisition, Project administration; **AS:** Conceptualisation, Methodology, Investigation, Writing – Review and Editing, Approved the manuscript**; KC:** Formal analysis, Visualisation, Approved the manuscript; **AO:** Investigation, Approved the manuscript**; GS:** Investigation, Approved the manuscript**; AZ:** Investigation, Approved the manuscript**; AEr:** Investigation, Approved the manuscript**; AEl:** Investigation, Approved the manuscript**; BH**: Investigation, Approved the manuscript**; AM‐F:** Sample collection and clinical information research, Approved the manuscript; **AT:** Investigation, Approved the manuscript**; JR‐G:** Sample collection and clinical information, Writing – Review and Editing, Approved the manuscript; **PK:** Sample collection and clinical information research, Supervision, Writing – Review and Editing, Approved the manuscript; **MM:** Resources, Supervision, Funding acquisition, Approved the manuscript; **AK:** Supervision, Funding acquisition, Writing – Review and Editing, Approved the manuscript.

## Supporting information


**Table S1.** Primer sequences, siRNA sequence, and WT‐ and ED‐miR‐200b‐3p sequences.
**Table S2.** A‐to‐I edited sites detected in HGSOC patients and healthy individuals.
**Table S3.** List of potential targets of edited miR‐200b‐3p.
**Fig. S1.** Quantitative assessment of transfected miR‐200b‐3p and ADAR1 expression amount in OVCAR3 and CAOV3 cell lines.
**Fig. S2.** *ADAR1* knockdown reduces cell proliferation, colony formation, migration, and 3D growth.
**Fig. S3.** Global changes in the transcriptome profile in transfected OVCAR3 and CAOV3 cell lines.
**Fig. S4.** Effect of miR‐200b‐3p editing on caspase 3/7 activity.
**Fig. S5.** Functional annotation analysis of predicted mRNA‐target genes.
**Fig. S6.** The sequence of the *MXI1* gene with the putative binding sites of ED‐miR‐200b‐3p.

## Data Availability

The human sequence data generated in this study are not publicly available due to patient privacy requirements but are available upon reasonable request from the corresponding author. Other data generated in this study are available within the article and its Supporting Information files.
