# Supplementary material for: Adenosine‐to‐inosine editing of miR‐200b‐3p is associated with the progression of high‐grade serous ovarian cancer
Source: Mol Oncol. 2025 Aug 8;20(2):409–27. doi: 10.1002/1878-0261.70106 (PMC12936423; doi:10.1002/1878-0261.70106)
Supplement: Supplementary file 1 — Table S1. Primer sequences, siRNA sequence, and WT‐ and ED‐miR‐200b‐3p sequences. Table S2. A‐to‐I edited sites detected in HGSOC patients and healthy individuals. Table S3. List of potential targets of edited miR‐200b‐3p. Fig. S1. Quantitative assessment of transfected miR‐200b‐3p and ADAR1 expression amount in OVCAR3 and CAOV3 cell lines. Fig. S2. ADAR1 knockdown reduces cell proliferation, colony formation, migration, and 3D growth. Fig. S3. Global changes in the transcriptome profile in transfected OVCAR3 and CAOV3 cell lines. Fig. S4. Effect of miR‐200b‐3p editing on caspase 3/7 activity. Fig. S5. Functional annotation analysis of predicted mRNA‐target genes. Fig. S6. The sequence of the MXI1 gene with the putative binding sites of ED‐miR‐200b‐3p. [file MOL2-20-409-s001.pdf]

Supplementary Material

Adenosine-to-inosine editing of miR-200b-3p is associated with the progression of high-grade serous ovarian cancer

Magdalena Niemira[1*], Anna Skwarska[2], Karolina Chwiałkowska[3], Agnieszka Ostrowska[1], Gabriela Sokołowska[1], Anna Zeller[1], Anna Erol[1], Andrzej Eljaszewicz[4], Bartosz Hanczaruk[4], Anna Michalska-Falkowska[5], Agnieszka Tarasik[5], Joanna Reszec-Gielazyn[5,6], Pawel Knapp[7], Marcin Moniuszko[4], Adam Kretowski[1]

*** Correspondence:** Corresponding Author: magdalena.niemira@umb.edu.pl

Supplemental Tables:

Table S1. Primer sequences, siRNA sequence, and WT- and ED-miR-200b-3p sequences.

Table S2. A-to-I edited sites detected in HGSOC patients and healthy individuals.

Table S3. List of potential targets of edited miR-200b-3p.

Supplemental Figures:

Figure S1. Quantitative assessment of transfected miR-200b-3p and ADAR1 expression amount in OVCAR3 and CAOV3 cell lines.

Figure S2. *ADAR1* knockdown reduces cell proliferation, colony formation, migration, and 3D growth.

Figure S3: Global changes in the transcriptome profile in transfected OVCAR3 and CAOV3 cell lines.

Figure S4. Effect of miR-200b-3p editing on caspase 3/7 activity.

Figure S5. Functional annotation analysis of predicted mRNA-target genes.

Figure S6. The sequence of the *MXI1* gene with the putative binding sites of ED-miR-200b-3p.

Table S1. Primer sequences, siRNA sequence, and list of Assay ID (Qiagen, Germany) with miRNA sequences.

**Primer**	**Used for:**	**Sequence (5' to 3')**
ADAR1_forward	qPCR	TGTAAAACGACGGCCAGT
ADAR1_reverse	qPCR	CAGGAAACAGCTATGACC
MXI1_forvard	qPCR	TGTAAAACGACGGCCAGT
MXI1_reverse	qPCR	CAGGAAACAGCTATGACC
ZEB1_forvard	qPCR	GCCAATAGACCAGACAGTGTT
ZEB1_reverse	qPCR	TTTGGCTGGATCACTTTCAAG
GAPDH_forward	qPCR	TGCACCACCAACTGCTTAGC
GAPDH_reverse	qPCR	GGCATGGACTGTGGTCATGAG
**siRNA**	**Targets**	**Sequence (5' to 3')**
siADAR1	ADAR1	GAGAUUCUCUCAGCCUAAA(dT)(dT)
**miRNA**	**Assay ID**	**Sequence (5' to 3')**
WT-miR-200b-3p	YP00206071	UAAUACUGCCUGGUAAUGAUGA
ED-miR-200b-3p	YCP0059764	UAAUGCUGCCUGGUAAUGAUGA
miR-103-3p	YP00204063	AGCAGCAUUGUACAGGGCUAUGA
miR-199b-5p	YP00204152	CCCAGUGUUUAGACUAUCUGUUC

Table S2. A-to-I edited sites were detected in HGSOC patients and healthy individuals. The edited adenosine within the mature miRNA sequence is in bold font; the seed sequence is underlined; miRNA: microRNA.

miRNA ID	Position	Sequence	log2FC (A-to-I %)	FDR
miR-200b-3p	5	UAAU**A**CUGCCUGGUAAUGAUGA	1.34	3.21E-04
miR-411-5p	5	UAGU**A**GACCGUAUAGCGUACG	0.82	1.03E-03
miR-381-3p	4	UAU**A**CAAGGGCAAGCUCUCUGU	0.55	1.03E-03
miR-148b-5p	13	AAGUUCUGUUAU**A**CACUCAGGC	-0.42	4.81E-03
miR-376c-3p	6	AACAU**A**GAGGAAAUUCCACGU	-2.29	6.05E-02
miR-421	14	AUCAACAGACAUU**A**AUUGGGCGC	-0.29	1.32E-01
miR-27a-5p	1	**A**GGGCUUAGCUGCUUGUGAGCA	-0.55	1.43E-01
miR-3157-3p	13	CUGCCCUAGUCU**A**GCUGAAGCU	-1.83	2.84E-01
miR-497-5p	2	C**A**GCAGCACACUGUGGUUUGU	0.11	3.97E-01
miR-1301-3p	5	UUGC**A**GCUGCCUGGGAGUGACUUC	-0.17	3.97E-01
miR-339-3p	15	UGAGCGCCUCGACG**A**CAGAGCCG	0.23	4.39E-01
miR-3622a-3p	3	UC**A**CCUGACCUCCCAUGCCUGU	0.37	5.60E-01
miR-641	3	AA**A**GACAUAGGAUAGAGUCACCUC	0.15	6.20E-01

Table S3. List of potential targets of edited miR-200b-3p.

Gene ID	logFC	FDR	Seed Location	Target score
MXI1	**-1.55**	**5.74E-19**	**882, 1024, 1699**	**94**
TSHZ3	-1.64	9.97E-17	1575	84
F3	-1.14	6.65E-14	171	74
OTX1	-1.14	5.07E-13	1206	50
RC3H2	-1.13	3.52E-12	166	80
TRAM1	-1.11	9.69E-12	59,138	92
UBXN2B	-1.08	1.08E-02	68, 364, 3000	92
PAQR3	-1.05	1.55E-02	883, 1091, 2147	68
DIXDC1	-1.03	2.09E-02	921, 3247	93
RAB5C	-1.03	4.07E-02	553, 564	92

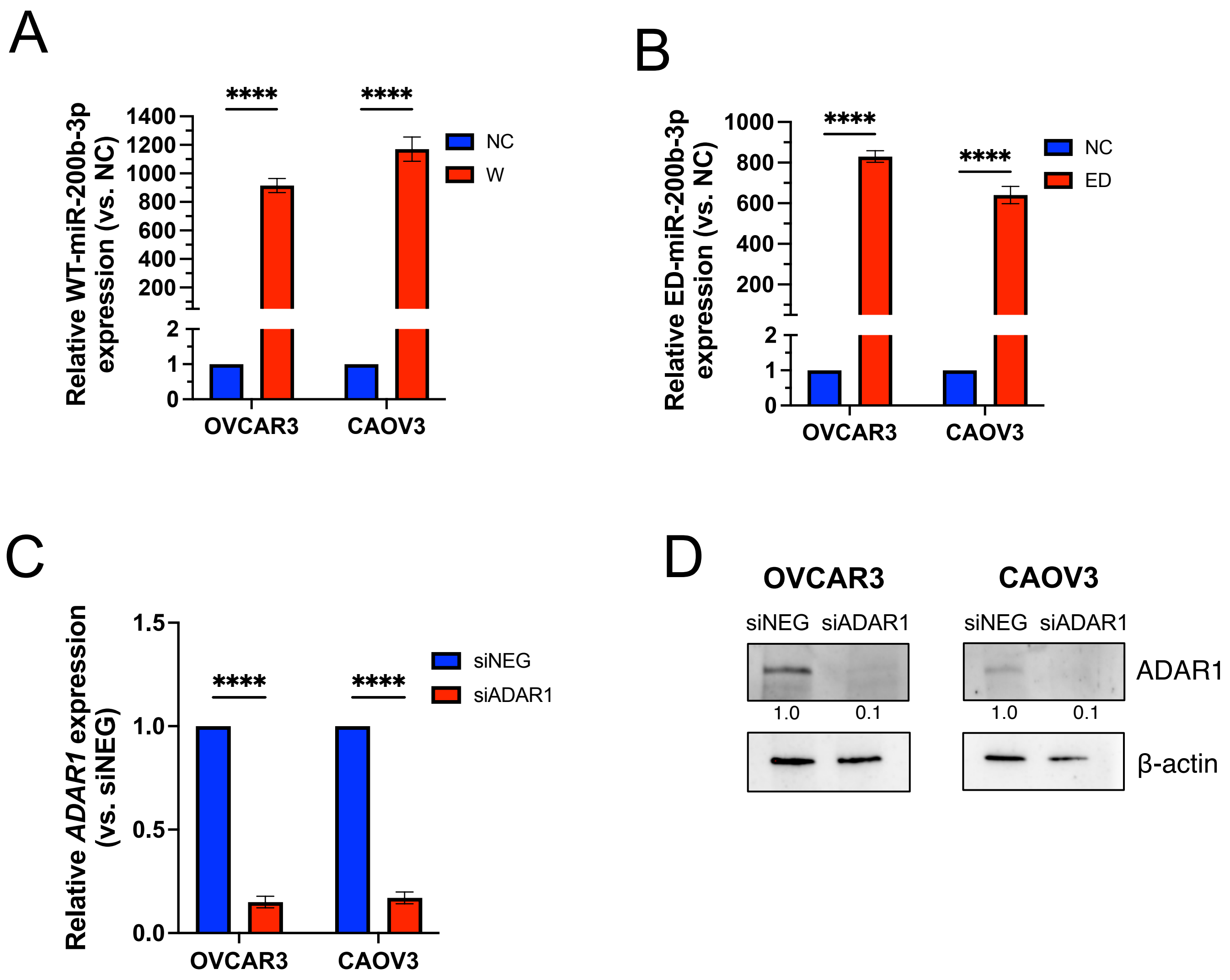

Figure S1. Quantitative assessment of transfected miR-200b-3p and *ADAR1* expression amount in OVCAR3 and CAOV3 cell lines. (A) The overexpression amount of unedited miR-200b-3p (WT miR-200b-3p) upon 24 h transfection with 50 nM wide-type miR-200b-3p mimics in OVCAR3 and CAOV3 cell lines by qRT-PCR. Results are mean ± SD, n = 3. Significance: Student's *t* test analysis; ****$P < 0.0001$. (B) The overexpression amount of edited miR-200b-3p upon 24 h transfection with 50 nM edited miR-200b-3p mimics in OVCAR3 and CAOV3 cell lines by qRT-PCR. Results are mean ± SD, n = 3. Significance: Student's *t* test analysis; ****$P < 0.0001$. (C) Knockdown effect of *ADAR1* by siRNA validated by qRT-PCR upon 24 h transfection with siADAR1. Results are mean ± SD, n = 3. Significance: Student's *t* test analysis; ****$P < 0.0001$. (D) The level of ADAR1 in OVCAR3 and CAOV3 cells transfected with 50 nM siNEG (negative control) and siADAR1 for 24 h was analyzed by Western blotting. Bands were analyzed by densitometry using ImageJ software and normalized to β-actin.

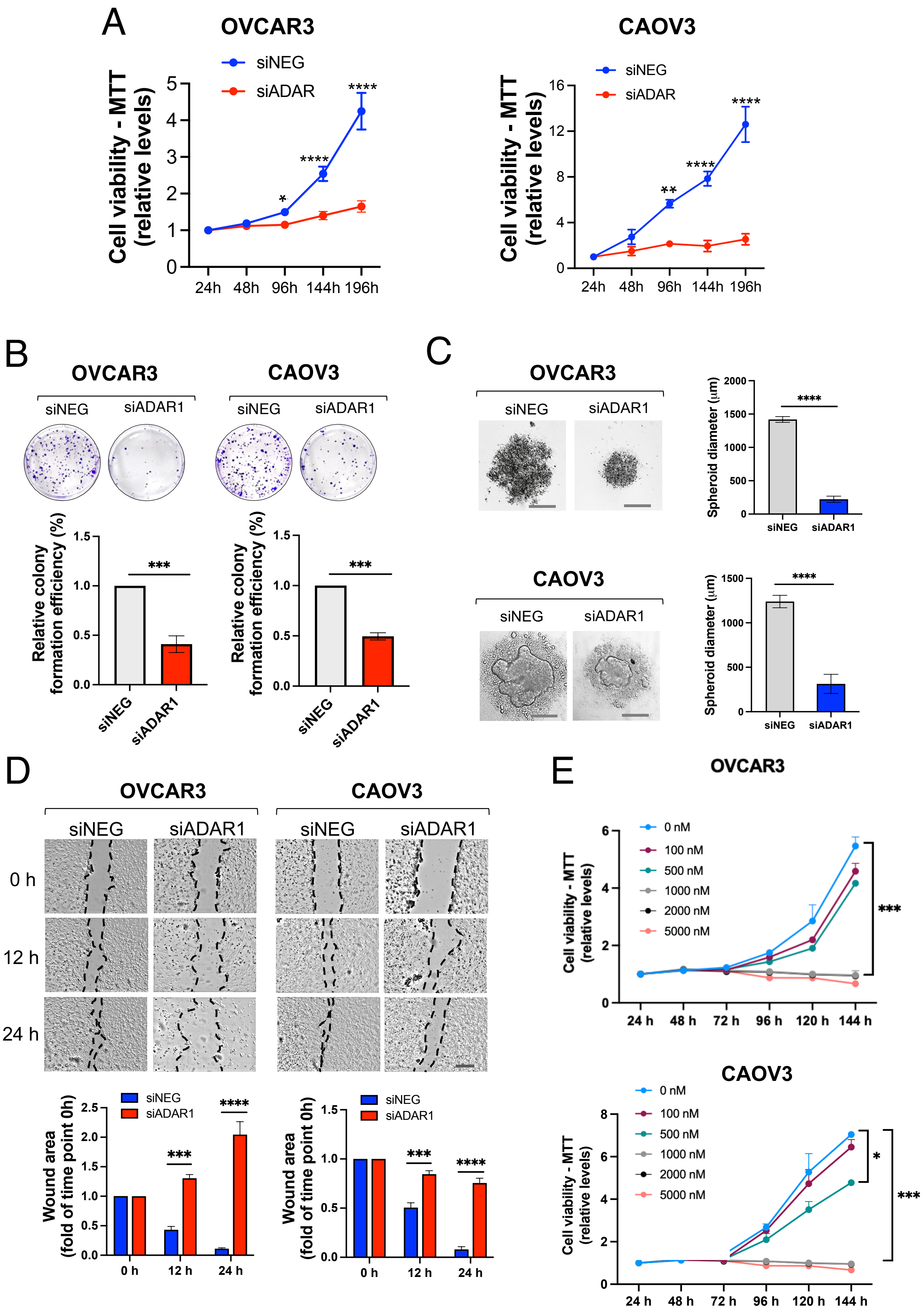

A
OVCAR3
siNEG
siADAR
Cell viability - MTT (relative levels)
24h 48h 96h 144h 196h
CAOV3
B
Relative colony formation efficiency (%)
siNEG siADAR1
C
Spheroid diameter (μm)
D
0 h
12 h
24 h
Wound area (fold of time point 0h)
E
0 nM
100 nM
500 nM
1000 nM
2000 nM
5000 nM
24 h 48 h 72 h 96 h 120 h 144 h

Figure S2. *ADAR1* knockdown reduces cell proliferation, colony formation, migration, and 3D growth. (A) MTT assay at the indicated time points. Results are mean ± SD, n = 3. Significance: Two-way ANOVA; $^{**}P < 0.01$, $^{****}P < 0.0001$. (B) Upper panel: Representative images of crystal violet-stained colonies. Bottom panel: quantitative analysis of colony formation assay. Results are mean ± SD, n = 6. Significance: Student's *t* test analysis; $^{***}P < 0.001$, $^{****}P < 0.0001$. (C) The left panel shows representative images of 3D spheroids cultures of cells transfected with siDAR1 and siNEG. Scale bars = 500 μm. The right panel shows changes in spheroids' diameter. Results are mean ± SD, n = 3. Significance: Student's *t* test analysis; $^{****}P < 0.0001$. (D) Representative bright-field images show that the acceleration of gap closure varied between cells transfected with siADAR1 and siNEG. Wound healing expressed as the remaining area uncovered by the cells was calculated using ImageJ software. Scale bar = 100 μm. Results are mean ± SD, n = 3. Significance: Student's *t* test analysis; $^{***}P < 0.001$, $^{****}P < 0.0001$. (E) MTT assay as the indicated time points at various concentrations of 8-azaadenosine (8-aza), the inhibitor of A-to-I editing. Results are mean ± SD, n = 3; $^{*}P < 0.05$, $^{***}P < 0.001$.

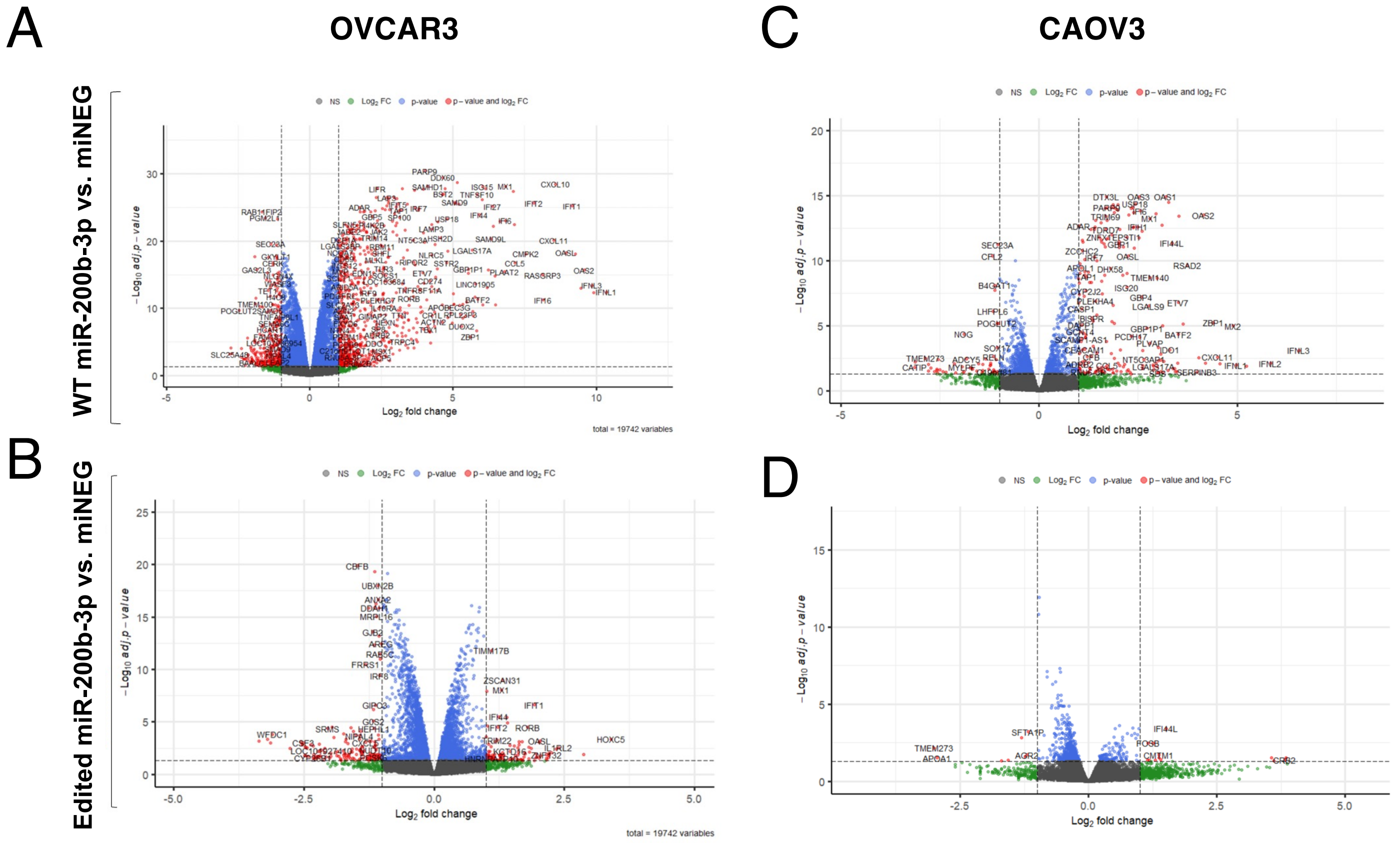

Figure S3. Global changes in the transcriptome profile in (A) OVCAR3 cells transfected with 50 nM WT-miR-200b-3p for 24 h, (B) OVCAR3 cells transfected with 50 nM ED-miR-200b-3p for 24 h, (C) CAOV3 cells transfected with 50 nM WT-miR-200b-3p for 24 h, and (D) CAOV3 cells transfected with ED-miR-200b-3p for 24 h (n = 3). The horizontal line is at a false discovery rate (FDR) = 0.05, and the vertical line is at |log2FC| = 1.

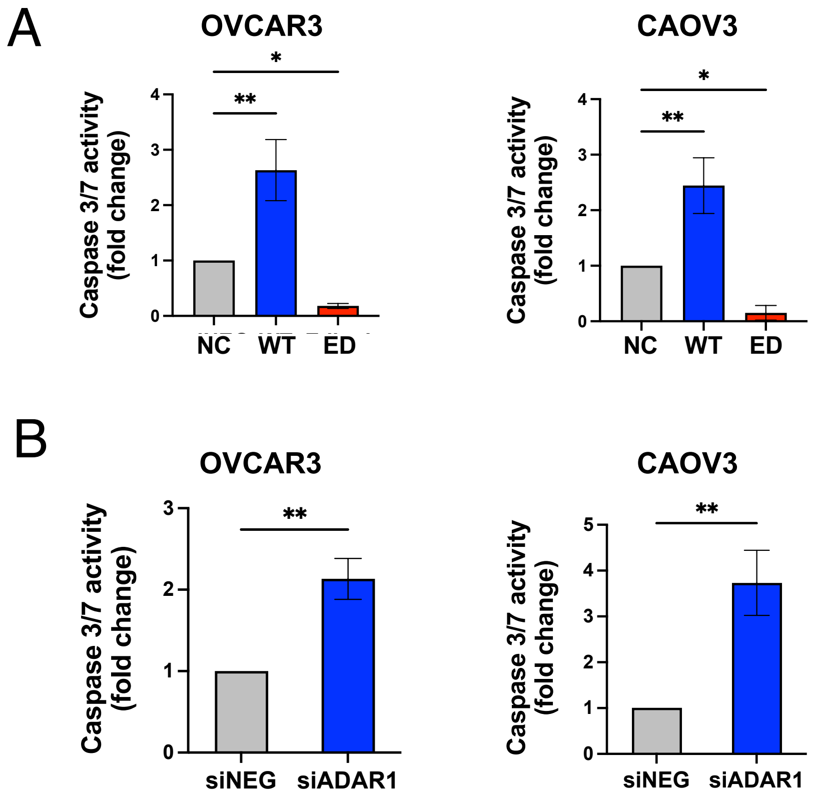

Figure S4. Relative caspase 3/7 activity in (A) OVCAR3 and CAOV3 transfected with 50 nM ED-miR-200b-3p and WT-miR-200b-3p mimics, and negative control (NC) for 24 h, and (B) 50nM siADAR1 and siNEG (siControl) for 24 h. Results are mean ± SD, n = 3. Significance: Student's *t* test analysis; *$P < 0.05$, **$P < 0.01$.

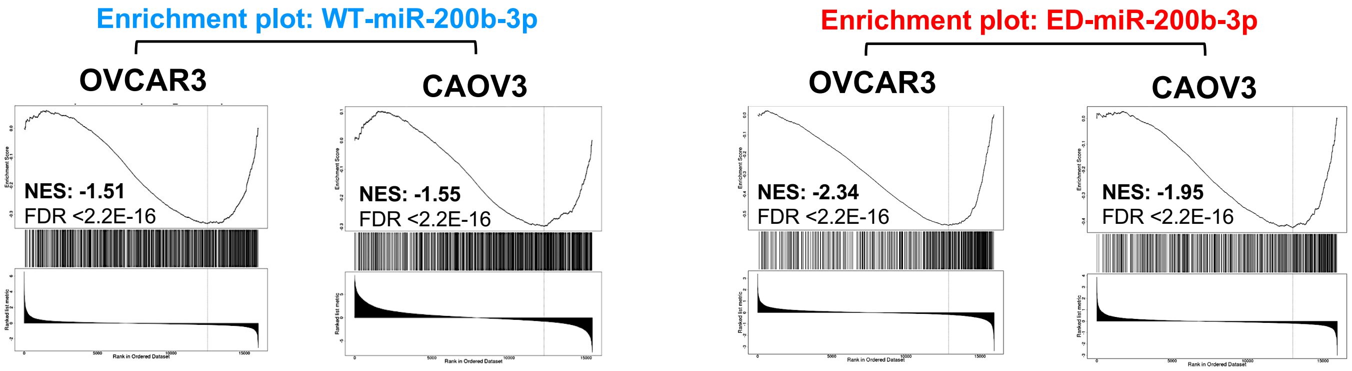

Figure S5. Enrichment of miRDB-predicted WT-miR-200b-3p and ED-miR-200b-3p putative targets in DEGs in OVCAR3 and CAOV3 cell lines upon transfection of 50 nM WT-miR-200b-3p and ED-miR-200b-3p for 24h.

MXI1 (ED-miR-200b-3p binding site)

1 aacccagcat gacataacag tgcagggcaa aatattcact gggccaattc aatacaaaca
61 atctcttaaa ttgggttcat gatgcagtct cctctttaaa acaaaacaaa acaaaacaaa
121 actatacttg aacaaaaggg tcagaggacc tgtatttaag caaatactta gcaaaaagtg
181 gggcagagcc tcccaaggag aacaaatatt cagaatattc atattggaaa aatcacaatt
241 tttaatggca gcagaaaact tgtgtgaaat tttcttgatt tgagttgatt gagaagagga
301 cattggagat gccatcctct ttctcttttc tagtttgctc atactacatt gagtagacac
361 atttaaggat ggggttatga acccttcctg agctttatgg tcctaaaagc aaaataaaaa
421 ctattcgaat gaaaagacaa gaaaatcagg tattaatctt ggatagctaa taatgagcta
481 ttaaaactca gcctgggaca gtttatcatg aagcctgtgg atgatcaatc ctttattatt
541 attttttttt tttgaaaaaa gctcatttca tgctctgcaa aaggagagac tcccatgaag
601 ccttttgaaa gggatcatca tgcagctcaa ctttctgttg gattccatgc taagcaagct
661 aaccttatcc tgcattgtta gcactaggca cccagctgcc acctctccat cctgctgccc
721 ttaggccaca tgggagcagt ccatgcatga cagcctctat cctacaaggc ctatgagtat
781 ggattggggg ggccaaaagg aaaaagctcc atgtgcctct ttgtctgcgt gggtcagaag
841 agttgtgcac gcagattagc aggccaaggt ctgagccaca g**cagcatt**tt tatttcagat
901 tttgataact gtttatatgt gttgaaaacc aaaatgacat ctttttaaag cttatccata
961 aaaaaaaata gatgtctttt atagtggaaa aacacatggg gaaaaaaatc atctattttg
1021 atg**cagcatt** tgataatgat aaaacacctc acacctcact ctttatagtg cacaaaatga
1081 atgaggtctg ggctaggtag aaaaagggtc aatgctattt ttgtttttag aatcattacc
1141 ttttaccagc ttttaaccat ctgatatcta tagtagacac actatcatag ttaacatagt
1201 aagttcagca cttgtctcat tttaatgtaa agatttgctt ccatttcct acaggcagtc
1261 tctctcttcc tcacagtccc actgtgcagg tgctattgtt actcttacga atattttcag
1321 taatgttatt ttcttctaag tgaaatttct agcctgcact ttgatgtcat gtgttccctt
1381 tgtctttcaa actccaaggt tcccttgtgg ccctctccct taccctggga aggcctcttg
1441 gagaccttac ccctggctgt ttggactttg tatactttaa ataatttaac tacccttaat
1501 tacttaaaaa aaaaaaaaag ctttatgatt ttcataactt attgctgatt ttaatggatt
1561 gttaatttca gtcctgtagt tttattttat gtttagatag ggctgggcaa ggaaaaagaa
1621 aataaagaca accatattta gcagtgcagt tgagttgtgt gttaatgtta gactatccct
1681 ttgtgagtga cactttaa**ca** **gcatt**cactg cttctatata tagtgtacca tcttggtcat
1741 acattacgcc tcaacatata cttgtgctct tcctttgcct ccagaagaag tttttccttg
1801 attgtgctat gtttcagtgg aagaaattct ttgaagtaga tgtgagtgaa aaactgcatg
1861 cctttagaag cccagtatca gaacttgcta cgtttcaggt gctagggact taatgaaaaa
1921 caggacaaaa caattccttt ttgtggccca ggtaaattat ttctggtttc acttataatt
1981 actaatggct gagtcaagat gttgtctctg tgtttgctta ctcttgatca agtgtgagac
2041 agtttgaaga ctgtgctacc atacaaagtg aatgaagcca gtgactaagc ttctgtttgt
2101 tttgttattc tcatggcctt cgcttgcatt atttgggcct tcattcagat gaacttgagg
2161 tgccattttg ttgcatatgt acaggattat gggctggaaa gcatttgtta taaacctata
2221 gtgcacattt taactgcccc ctaaattacc cttccctggg tttgttttcc ttggggtggt
2281 gtagattgta tgagtaagaa gtattaattt tttaaaagac aaatcaactt tgaagacaca
2341 aaagttaatt ggaagaaata aaaactgtga acgaagaa

Figure S6. The sequence of the *MXI1* gene with the putative binding sites (highlighted in blue) of ED-miR-200b-3p to the *MXI1* 3-UTR region, which was indicated using the mirDB database.
